# A novel inflammation-related signature for predicting prognosis and characterizing the tumor microenvironment in colorectal cancer

**DOI:** 10.18632/aging.204630

**Published:** 2023-04-02

**Authors:** Jinna Li, Jiapeng Yang, Rui Xing, Ying Wang

**Affiliations:** 1 Department of Oncology, Shengjing Hospital of China Medical University, Shenyang 110004, Liaoning, China; 2 Department of General Surgery, Shengjing Hospital of China Medical University, Shenyang 110004, Liaoning, China

**Keywords:** colorectal cancer, inflammation-related genes, tumor microenvironment, inflammatory response, TIMP1

## Abstract

Inflammation is a critical component of tumor progression, and it modifies the tumor microenvironment by various mechanisms. Here, we explore the effect of the inflammatory response on the tumor microenvironment in colorectal cancer (CRC). A prognostic signature consisting of inflammation-related genes (IRGs) was constructed and verified based on the inflammatory response by bioinformatics analysis. IRG risk model was identified as an independent prognostic factor in CRC, and was related to biological processes of extracellular matrix, cell adhesion and angiogenesis. The IRG risk score predicted the clinical benefit of ipilimumab. Weighted correlation network analysis identified TIMP1 as the hub gene of the inflammatory response in the IRG risk model. Coculture experiments with macrophages and CRC cells revealed that TIMP1 promoted macrophage migration, inhibited the expression of M1 markers (CD11C and CD80), and promoted the expression of M2 markers (ARG1 and CD163). TIMP1 promoted the expression of ICAM1 and CCL2 by activating the ERK1/2 signaling pathway to promote macrophage migration and M2-like polarization. These IRGs in the risk model regulated stromal and immune components in the tumor microenvironment and could serve as potential therapeutic targets in CRC. TIMP1 promoted macrophage migration and meditated macrophage M2 polarization by activating ERK1/2/CLAM1 and CCL2.

## INTRODUCTION

Colorectal cancer (CRC) is the third most common malignant tumor, and among cancers it has the second highest mortality worldwide [1]. Considerable progress has been made in comprehensive therapy over the past few decades, and effective treatments mainly include radical surgical resection, chemotherapy, radiotherapy, molecular targeting treatment and immune checkpoint inhibitor treatment. However, because of its late diagnosis and high recurrence rate, the prognosis of CRC is still poor, and the mortality rate remains high [2]. At present, cancer biology is shifting from a “cancer-cell-centric” concept to a more comprehensive view, placing tumor cells in a network of stromal components comprising inflammatory cells, immune cells, fibroblasts and vascular cells, which interact with each other and collectively form the tumor microenvironment (TME) [3].

Inflammation is a critical component of tumor progression, and it alters the TME by various mechanisms, such as the production of cytokines and proinflammatory mediators, angiogenesis, and tissue remodeling. CRC has long been considered one of the best examples of tumors closely related to chronic inflammation, which can occur in the earliest stages of tumorigenesis [4]. Numerous bacterial strains in the intestinal tract coexist harmoniously with their hosts; however, any substantial change in the bacterial population will lead to a considerable impact on the inflammatory response and promote the development of tumors [4]. In addition, cancer cells, along with their surrounding inflammatory cells and stromal cells, participate in harmonious interactions to form an inflammatory TME [5]. Inflammation has a significant influence on the constitution of the TME, especially the plasticity of both stromal and tumor cells [3]. In the process of tumor growth, inflammatory cells and their mediators can block potential antitumor immunity and facilitate tumor-supporting functions, such as stimulating angiogenesis and recruiting fibroblasts and other stromal cells [6]. As such, inflammation is now regarded as one of the core hallmarks of cancer [7].

Inflammasomes are macromolecular complexes that trigger central and rapid inflammatory responses to cytoplasmic damage [8]. Inflammasomes play contrasting roles in the intricate interaction between malignant tumor cells and their microenvironment [4]. They may play a role at the cell-autonomous level, eliminating precursors of malignancy through programmed cell death, or in turn, they may stimulate the production of trophic factors for cancer cells and the surrounding stroma [9]. In addition, NLRC4 participates in M2 polarization and IL-1β and VEGF production in tumor-associated macrophages (TAMs), which promote the growth of liver metastasis of CRC via inflammasomes [10]. The inhibition of inflammasomes or the neutralization of their products, mainly IL-1b and IL-18, has a significant influence on the occurrence and development of tumors [9].

This study constructed a robust inflammation-related gene (IRG) signature for CRC based on bioinformatics analysis. We explored the prognostic value of the IRG risk score and its relationship with clinicopathological factors and molecular features. In addition, we identified the biological processes and signaling pathways in which the IRGs were involved. Moreover, we investigated the associations of different cell populations with the IRG risk score in the TME. Finally, tissue inhibitor of matrix metalloproteinase-1 (TIMP1) was confirmed to perform a key function in the inflammatory response of CRC and could promote macrophage migration. Taken together, the findings of this research will help to understand the mechanisms of the inflammatory response in CRC and guide more precise prognosis prediction and personalized therapy.

## RESULTS

### Construction of the IRG risk score in CRC

**Figure 1:**
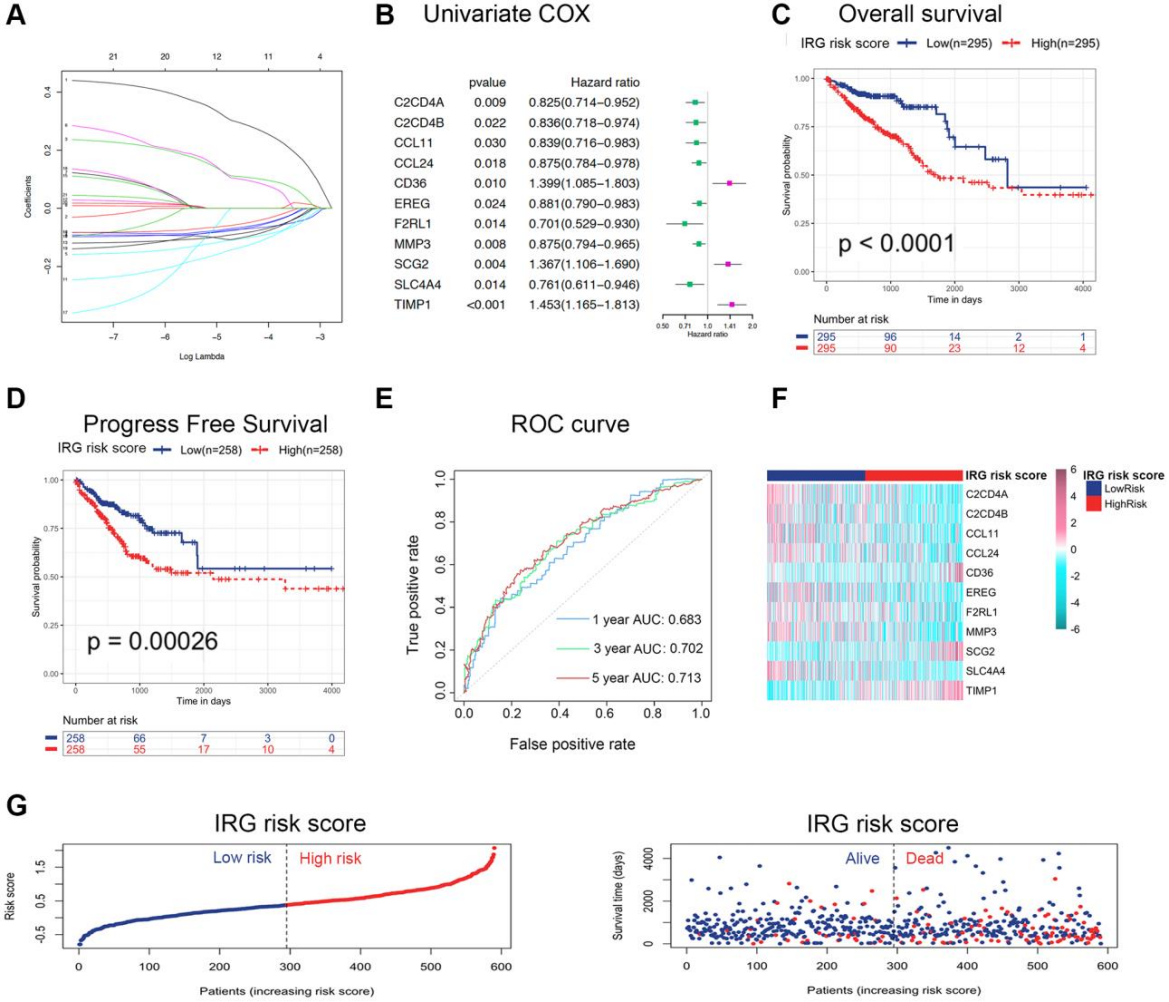
**Construction and validation of the IRG risk score in CRC.** (**A**) LASSO regression was performed, and the minimum criteria and coefficients were calculated. (**B**) Univariate Cox regression analysis of 11 candidate prognosis-related IRGs. (**C**, **D**) Kaplan-Meier analysis of overall survival (**C**) and progression-free survival (**D**) based on the IRG risk score of CRC patients in the TCGA dataset (log-rank *p* < 0.0001). (**E**) ROC curve of the IRG risk score for 1-, 3- and 5-year overall survival in the TCGA dataset. (**F**, **G**) The distribution of risk scores, survival status and candidate gene expression data of CRC patients based on the IRG risk score in the TCGA dataset.

To explore the clinical role of inflammatory status in CRC, an IRG set containing 931 genes, including inflammatory response genes (gene set M5932, M10617 and M15261 extracted from the Molecular Signatures Database (MSigDB)) and inflammasome-related genes, were summarized in Supplementary Table 1 [8]. After performing univariate Cox regression analysis, 76 genes were correlated with the survival of CRC (*p* < 0.05). Furthermore, differentially expressed genes (DEGs) between nontumor and tumor tissues were identified by the R package “limma” (adjusted *p* < 0.05 and |logFC| > 1.0). We identified 136 DEGs, including 52 upregulated genes and 84 downregulated genes, among the IRGs (Supplementary Figure 1A), and found 21 differentially expressed IRGs with univariate Cox *p* values < 0.05 (Supplementary Table 2 and Supplementary Figure 1B). According to the above results, least absolute shrinkage and selection operator (LASSO) regression analysis was applied at 1000 maxit in The Cancer Genome Atlas (TCGA) dataset (Figure 1A), and we identified eleven IRGs as potential risk-related genes in the prognostic signature. Among these eleven genes, C2CD4A, C2CD4B, CCL11, CCL24, EREG, F2RL1, MMP3 and SLC4A4 were defined as protective genes with hazard ratios (HRs) < 1, whereas CD36, SCG2 and TIMP1 were defined as risk-conferring genes with HRs > 1 (Figure 1B). Next, the expression of these eleven genes was used to predict the risk level of the inflammatory response in CRC. The formula was as follows: IRG risk score = (−0.1029 × C2CD4A expression) + (−0.0514 × C2CD4B expression) + (−0.0986 × CCL11 expression) + (−0.0845 × CCL24 expression) + (0.0976 × CD36 expression) + (−0.0694 × EREG expression) + (−0.0636 × F2RL1 expression) + (−0.0708 × MMP3 expression) + (0.1073 × SCG2 expression) + (−0.1303 × SLC4A4 expression) + (0.2884 × TIMP1 expression). The patients were classified into low-risk and high-risk groups based on the median IRG risk score.

### The IRG risk score was a robust prognostic indicator

To assess the prognostic value of the IRG risk score, we applied the risk formula in the TCGA dataset (discovery cohort) and GSE39582 dataset (validation cohort) and stratified patients according to the median IRG risk score. The Kaplan-Meier survival analyses in the TCGA and GSE39582 datasets showed that overall survival (OS) and progression-free survival (PFS) in the high-risk group were both significantly shorter than those in the low-risk group (Figure 1C, 1D and Supplementary Figure 1C, 1D). To investigate whether the IRG risk score was an independent prognostic factor for CRC, we performed univariate and multivariate Cox regression analyses in the TCGA dataset and GSE39582 dataset. The univariate analysis showed that five risk factors, including IRG risk score, pathologic stage, pathologic T stage, pathologic N stage, and pathologic M stage, were correlated with unfavorable survival in the two cohorts (Supplementary Table 3). KRAS and BRAF mutation status were not correlated with OS in the univariate Cox regression analysis. TP53 mutation status was significantly relevant to survival in the TCGA dataset (*p* < 0.05), while the correlation was not significant in the GSE39582 dataset. The multivariate Cox regression analysis of the above five risk factors suggested that the IRG risk score was an independent prognostic factor in CRC (Table 1 and Supplementary Table 4). Overall, the IRG risk score was an independent predictive factor in CRC, which highlighted its potential as a prognostic marker for malignant progression in CRC.

**Table 1:** Univariate and multivariate Cox regression analyses in the TCGA dataset.

**Variable**	**Univariate regression**			**Multivariate regression**		
	**HR**	**95% CI**	***p* Value**	**HR**	**95% CI**	***p* Value**
Risk score	3.8334	2.3288–6.3101	<0.0001	3.1620	1.8287–5.4670	<0.0001
Pathologic_stage	2.0305	1.5438–2.6708	<0.0001	1.4240	0.6594–3.0760	0.3680
Pathologic_T	2.5614	1.5665–4.1880	0.0002	1.5450	0.8559–2.7900	0.1490
Pathologic_N	1.9038	1.4319–2.5313	<0.0001	1.0040	0.6064–1.6610	0.9890
Pathologic_M	3.2602	1.9388–5.4824	<0.0001	1.6760	0.5772–4.8670	0.3420

### The IRG risk score predicted survival time

**Figure 2:**
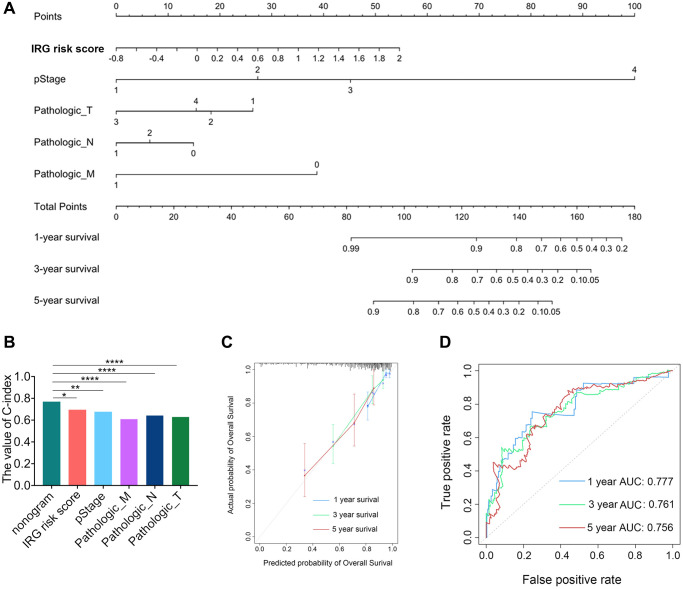
**Development and validation of the IRG risk score nomogram.** (**A**) Development of the IRG risk score nomogram. (**B**) The C-index value of the IRG nomogram was significantly higher than the C-index of other constitutive factors. (**C**) The calibration plot exhibited wonderful agreement between prediction and observation in the probabilities of 1-, 3- and 5-year overall survival. (**D**) Receiver operating characteristic (ROC) curve of the IRG nomogram for 1-, 3- and 5-year overall survival in the TCGA dataset.

To assess the prognostic validity of the IRG risk score in CRC, time-dependent receiver operating characteristic (ROC) analysis was performed based on 1-, 3- and 5-year survival, and the respective area under the curve (AUC) values were 68.3%, 70.2% and 71.3% in the TCGA dataset (Figure 1E), which indicated its accuracy in predicting survival at these intervals. The ROC analysis in the GSE39582 dataset reached the same results (Supplementary Figure 1E). The heatmap revealed the mRNA expression patterns of these eleven IRGs in the high- and low-risk groups (Figure 1F and Supplementary Figure 1F). The risk curve and scatter plot demonstrated that patients in the high-risk group showed poorer prognoses, whereas patients in the low- risk group had more favorable prognoses (Figure 1G and Supplementary Figure 1G). Chemotherapy was the main treatment strategy for CRC patients. We conducted survival analyses to examine whether the IRG risk score could serve as a marker for predicting the response to chemotherapy. CRC patients receiving chemotherapy in the low-risk group had longer OS than those in the high-risk group. Simultaneously, no difference in OS was found between the nonchemotherapy group and chemotherapy group with a high IRG risk score, which showed that the IRG risk score may participate in mediating chemotherapy resistance in CRC (Supplementary Figure 1H). In addition, the low IRG risk score group had longer PFS than the high-risk group in patients receiving chemotherapy (Supplementary Figure 1I). Moreover, we established nomograms in the TCGA and GSE39582 datasets to achieve better predictive accuracy and translational potential (Figure 2A and Supplementary Figure 2A). The C-index of the nomogram was 0.7678, which was significantly higher than that of its constituent factors (IRG risk score: 0.6941, pathologic stage: 0.6757, pathologic T stage: 0.6284, pathologic N stage: 0.6430, and pathologic M stage: 0.6092) in the TCGA dataset (Figure 2B). The calibration plot showed a high degree of consistency between the predicted probability and actual 1-, 3- and 5-year survival rates (Figure 2C and Supplementary Figure 2B). The above results suggested that the IRG risk score could predict survival time for CRC patients. To estimate the validity of the nomogram for predicting survival, ROC curves were generated based on the 1-, 3- and 5-year survival rates, and the respective AUC values were 77.7%, 76.1% and 75.6% in the TCGA dataset (Figure 2D). In the validation dataset (GSE39582 dataset), we validated these results (1-year AUC: 74.2%, 3-year AUC: 73.3%, 5-year AUC: 68.5%) (Supplementary Figure 2C).

### The IRG risk score demonstrated a subtype expression preference and was validated as an independent prognostic factor in CRC

To explore the association between clinical parameters and the IRG risk score, patients were stratified into different subgroups. The expression analysis showed that the IRG risk score was higher in the stage III-IV subgroup, T3-4 subgroup, N1-2 subgroup, M1 subgroup, mutant-type BRAF subgroup and venous invasion subgroup than in each corresponding subgroup (Supplementary Figure 3A–3F). Patients with mutant-type KRAS in stage IV or with wild-type TP53 in stage I had significantly higher IRG risk scores than their corresponding subgroups (Supplementary Figure 3G, 3H). Furthermore, a stratified analysis was performed on the basis of the above clinical features to better assess the prognostic ability of the IRG risk score. The stratified analysis showed that the IRG risk score could be well applied to subgroups stratified by TNM stage (I-II, Supplementary Figure 4A; III-IV, Supplementary Figure 4B), N stage (N0, Supplementary Figure 4C; N+, Supplementary Figure 4D), and venous invasion (yes, Supplementary Figure 4E; no, Supplementary Figure 4F). When the prognostic risk was assessed on the basis of the IRG risk score, high-risk patients in each of these subgroups had worse survival than low-risk patients, which indicated that the IRG risk score could be a potential predictor of the malignant phenotype in CRC. In wild-type BRAF patients, high-risk group had worse survival than low-risk group (Supplementary Figure 4G), while there was no difference in survival between high- and low-risk groups in mutant-type BRAF patients (Supplementary Figure 4H). To further investigate the correlation between the IRG risk score and BRAF status in CRC, we compared wild-type BRAF patients with high scores and mutant-type BRAF patients in the TCGA dataset. The results showed no significant difference in OS, which indicated that the IRG risk score may have a risk level similar to that of BRAF mutation (Supplementary Figure 4I). Taken together, the IRG risk score demonstrated a subtype expression preference and was validated as an independent prognostic factor in CRC.

### The IRG risk score was associated with distinguishing genomic and transcriptomic spectra in CRC

To explore the molecular characteristics associated with the IRG risk score, we analyzed available mutation and copy number variation (CNV) information (Supplementary Figure 5A). The deletion events on chromosome 2p12 (LRRTM4), 11q22.3 (ACAT1 and CRYAB) and 12q13.1 (EMP1) were more common in the low-risk group. In addition, focal amplifications on chromosome 1q31.3 (LAMC1 and LAMC2), 9q34.3 (C8G and FCN1), 10q22.2 (ADK), 6q12 (PTP4A1 and PHF3), and 13q33.3 (COL4A1 and COLA4A2) were more common in the high-risk group (Supplementary Figure 5B). Then, we analyzed the distribution differences of somatic mutations between the low- and high-risk groups in the TCGA dataset using the “maftools” package. The low-risk group showed less tumor mutation burden (TMB) than the high-risk group, while some of the top ten most significantly mutated genes, such as APC, TP53 and FAT4, showed higher TMB in the low-risk group (Supplementary Figure 5C).

### The IRG risk score was associated with regulation of the extracellular matrix, cell adhesion, and angiogenesis in CRC

**Figure 3:**
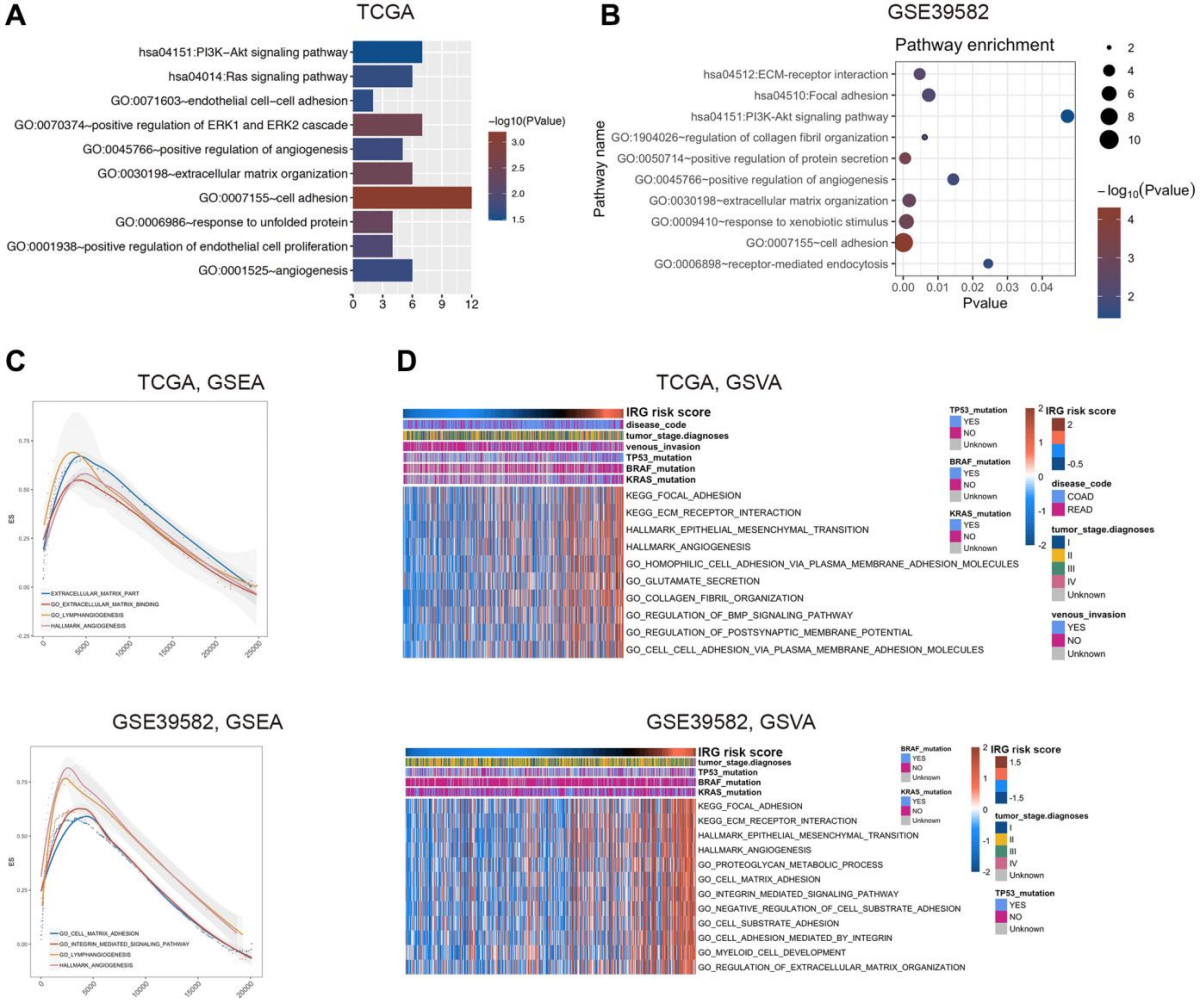
**Functional exploration of the IRG risk score in CRC.** (**A**, **B**) Gene Ontology (GO) and KEGG pathway analysis with IRG risk score-correlated genes in the TCGA (**A**) and GSE39582 (**B**) datasets. (**C**, **D**) Relative biological functions of these genes in the TCGA and GSE39582 datasets were verified by GSEA (**C**) and GSVA (**D**) analyses.

To elucidate the related signaling pathways and functions of the inflammatory response, we used correlated genes with the IRG risk score for functional enrichment analysis in the TCGA (|r|>0.4 and *p* < 0.05) and GSE39582 (|r|>0.5 and *p* < 0.05) databases, respectively (Supplementary Table 5). Gene Ontology (GO) and Kyoto Encyclopedia of Genes and Genomes (KEGG) pathway analysis of these genes by DAVID 6.8 showed that the IRG risk score was related to cell adhesion, extracellular matrix organization, positive regulation of angiogenesis and PI3K-AKT signaling pathway (Figure 3A, 3B). Consistent with the above results, gene set enrichment analysis (GSEA) showed that the enriched pathways related to the IRG risk score were extracellular matrix, cell adhesion, angiogenesis and lymphangiogenesis (Figure 3C). According to gene set variation analysis (GSVA), the IRG risk score was correlated with the following functional terms: extracellular matrix, focal adhesion, cell adhesion and angiogenesis (Figure 3D). In general, these results proved that the inflammatory process in the occurrence and development of CRC was mainly relevant to the biological processes of extracellular matrix, cell adhesion and angiogenesis, all of which are considered to be associated with the prognosis of CRC.

### The IRG risk score was associated with the stromal score, tumor purity, and microenvironment cell populations

**Figure 4:**
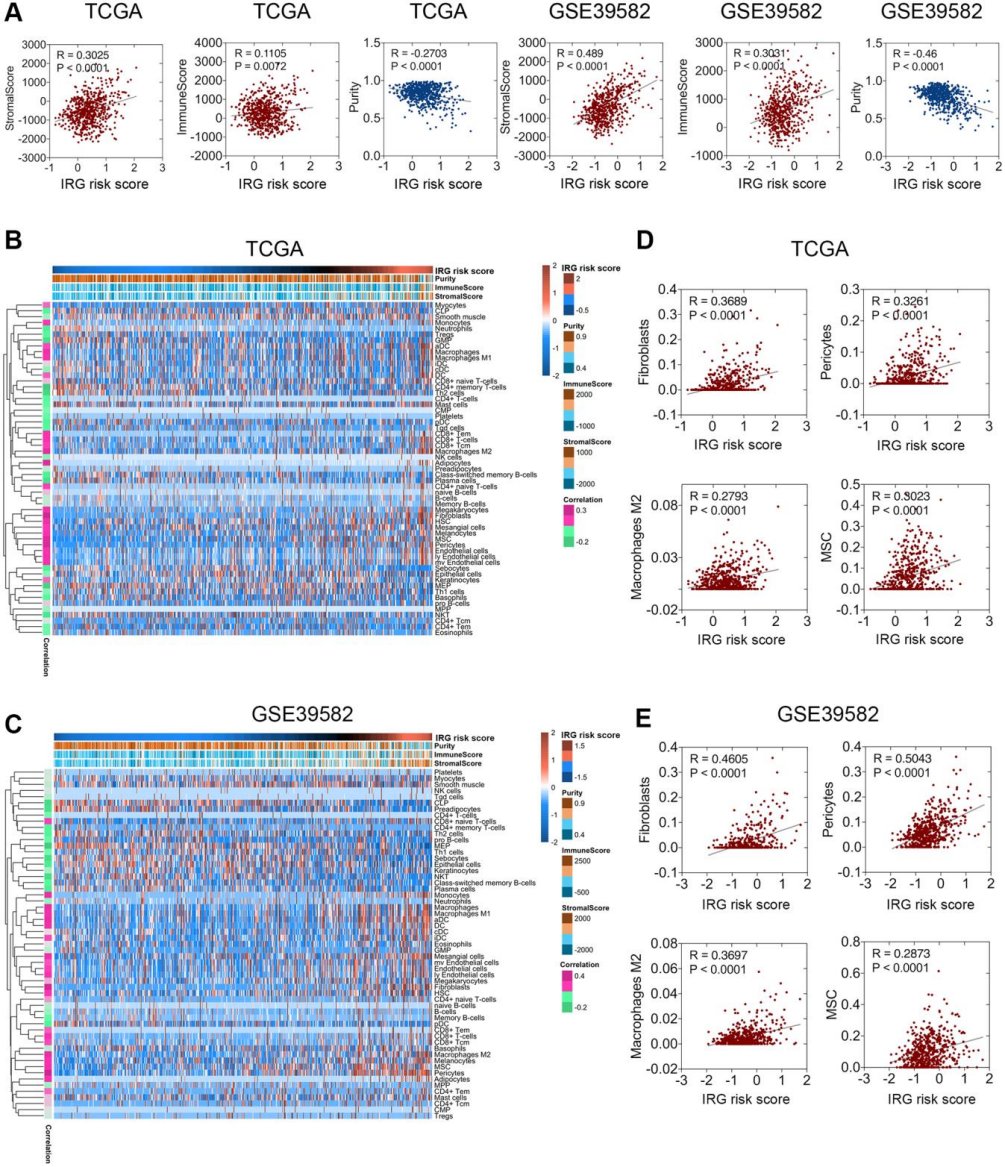
**Relationship between the IRG risk score and the CRC microenvironment.** (**A**) The IRG risk score was positively correlated with stromal score but negatively correlated with tumor purity in the TCGA and GSE 39582 datasets. (**B**, **C**) xCell analysis showed extreme enrichment of stromal and immune cells with high IRG risk scores. (**D**, **E**) IRG risk scores were positively correlated with fibroblasts, pericytes, M2 macrophages, and MSCs in the TCGA (**D**) and GSE39582 (**E**) datasets.

The above functional enrichment analysis demonstrated that the inflammatory response of CRC was associated with many biological processes of the stroma. However, the relationship between the inflammatory response and stroma in CRC has rarely been studied. Hence, we analyzed the relevance between the IRG risk score and stromal score, immune score, or tumor purity in CRC with the ESTIMATE package [11]. Pearson’s correlation analysis was applied to investigate the relevance between the TME indexes and the IRG risk score. The results showed that the IRG risk score was weakly positively correlated with stromal score but negatively correlated with tumor purity, while there was no stable correlation between the IRG risk score and immune score (Figure 4A). Among these factors, a closer relationship was found between the IRG risk score and stromal score, indicating that the inflammatory response mainly regulated the function of the extracellular matrix in CRC, which was consistent with the conclusion of the functional enrichment analysis. Further cell population enrichment analysis by xCell [12] revealed that the infiltration of dendritic cells, CD8+ T cells, adipocytes, lymphatic endothelial cells, M1 macrophages, M2 macrophages, mesenchymal stem cells (MSCs), and pericytes had a positive relevance to the IRG risk score, while the infiltration of megakaryocyte-erythroid progenitors (MEPs), CD4+ memory T cells, plasma cells, Th2 cells and natural killer (NK) T cells had a negative correlation with the IRG risk score (Figure 4B, 4C). Fibroblast, pericyte, M2 macrophage, and MSC enrichment were positively associated with the IRG risk score (Figure 4D, 4E). Collectively, these data showed that the IRG risk score was associated with the regulation of the local immune response and extracellular matrix in CRC.

### CRC cell-intrinsic TIMP1 regulated the migration capabilities of macrophages and induced their phenotypic transition to M2 macrophages *in vitro*

**Figure 5:**
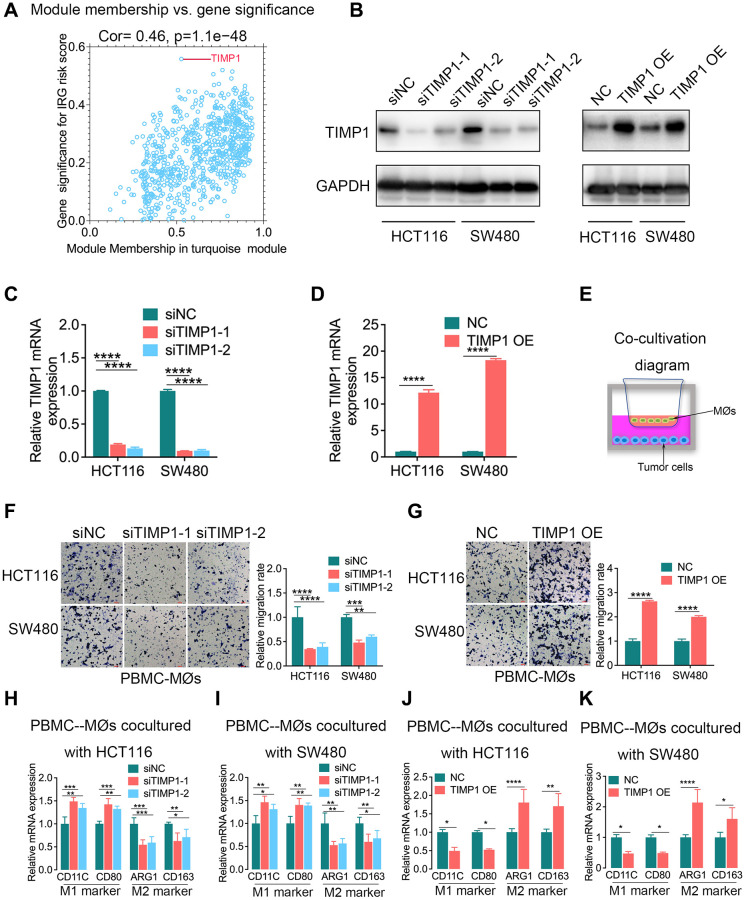
**TIMP1 was the hub gene of the inflammatory response and promoted macrophage infiltration and M2-like polarization in CRC.** (**A**) Scatterplot of gene significance for the IRG risk score and module membership in the turquoise module. (**B**–**D**) The knockdown and overexpression of TIMP1 were confirmed with Western blotting (**B**) and qPCR (**C** and **D**). (**E**) Diagram of colorectal cancer cells cocultured with macrophages. (**F**, **G**) The migration ability of macrophages was confirmed with Transwell assays in the TIMP1 knockdown group (**F**) and TIMP1 overexpression group (**G**). (**H**–**K**) PCR results of detecting the polarization of macrophages under different cocultures with CRC cells (Student’s *t* test or one-way ANOVA, *n* = 3). (Data are presented as the means ± standard deviations; ^*^indicates *P* < 0.05, ^**^indicates *P* < 0.01, ^***^indicates *P* < 0.001, and ^****^indicates *P* < 0.0001).

To identify the hub genes associated with the inflammatory response in CRC, we constructed a gene coexpression network using the weighted correlation network analysis (WGCNA) package. In this study, 3 was selected as the soft threshold power to exhibit the scale independence and degree of mean connectivity of the scale-free topology module (Supplementary Figure 6A). The heatmap plotted the topological overlap matrix (TOM) among 4575 genes in the analysis and indicated that each module was an independent validation, and a total of 14 modules were identified from the coexpression network (Supplementary Figure 6B). Module-trait relationships revealed that the turquoise module was identified as the key module for the highest correlation with the IRG risk score (r = 0.36, *p* = 3e-19), indicating that the genes involved in the turquoise module were most likely related to the inflammatory response in CRC (Supplementary Figure 6C). The turquoise module contained 907 genes. Scatterplots of gene significance (GS) and module membership (MM) were plotted in the turquoise module, and TIMP1 was identified as the inflammatory response-related hub gene that had the highest GS (Figure 5A).

TIMP1 modulates the pericellular proteolysis of a vast range of matrix and cell surface proteins, and affects tumor architecture and cell signaling [13]. The IRG risk score was found to be correlated with macrophages in a previous study (Figure 4B–4E). Therefore, we further explored the regulatory effect of TIMP1 on macrophages in CRC. The knockdown and overexpression of TIMP1 in HCT116 and SW480 cells were confirmed with Western blotting and qPCR (Figure 5B–5D). Then, we cocultured CRC cells, including HCT116 and SW480 cells, with peripheral blood mononuclear cells-derived macrophages (PBMC-MØs) (Figure 5E). The data revealed that TIMP1 knockdown CRC cells significantly decreased macrophage migration compared with the control group (Figure 5F), while TIMP1-overexpressing CRC cells promoted macrophage migration (Figure 5G). PCR assays in PBMC-derived macrophages cocultured with TIMP1 knockdown HCT116 or SW480 cells also confirmed increased M1 marker expression (CD11C and CD80; Figure 5H, 5I) and decreased M2 marker expression (ARG1 and CD163; Figure 5H, 5I), and corresponding results were obtained in PBMC-derived macrophages cocultured with TIMP1-overexpressing HCT116 or SW480 cells (Figure 5J, 5K).

### TIMP1 promoted the expression of ICAM1 and CCL2 by activating the ERK1/2 signaling pathway

**Figure 6:**
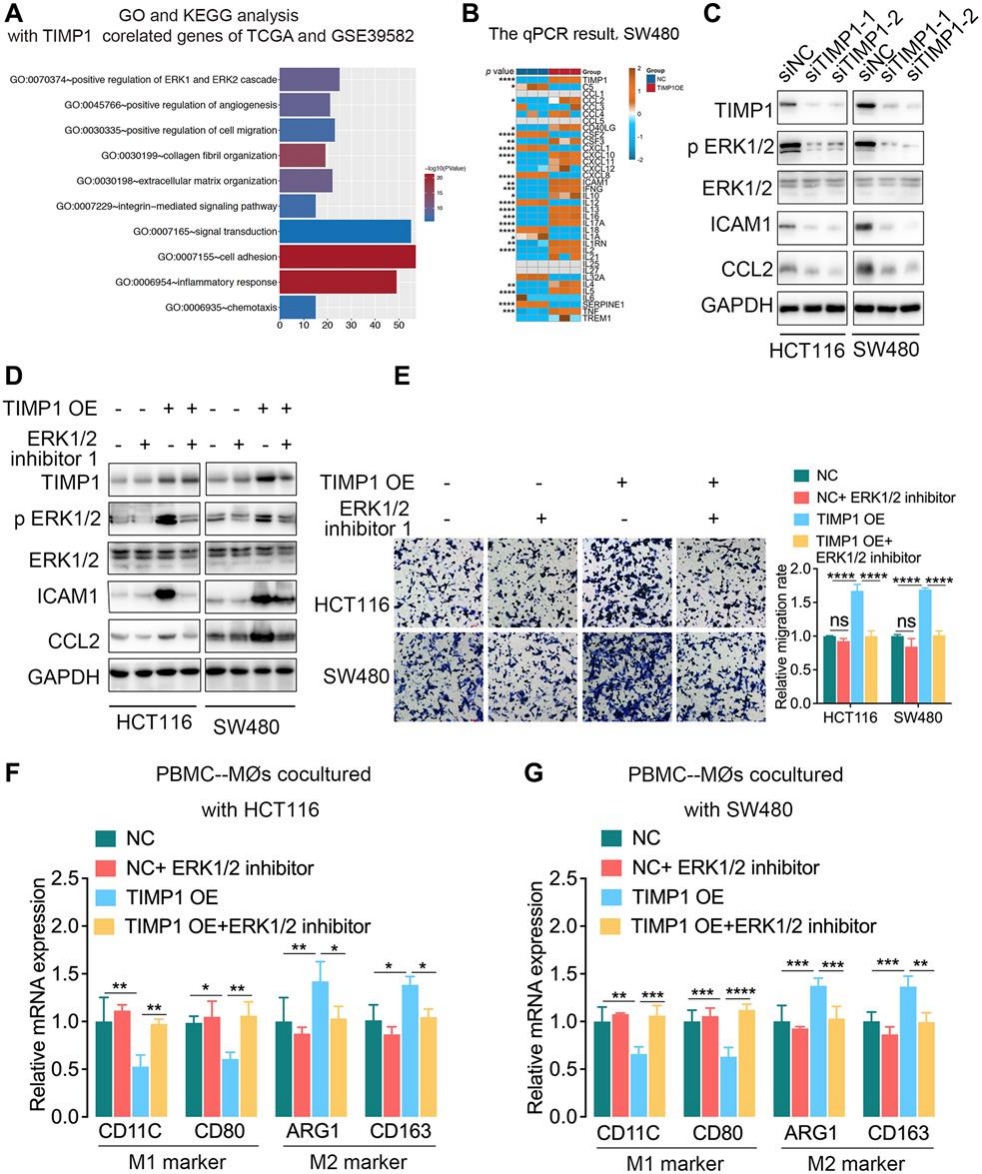
**TIMP1 promoted macrophage migration and M2-like polarization by activating the ERK1/2 pathway in CRC.** (**A**) GO analysis and KEGG pathway analysis of overlapping TIMP1-correlated genes in the TCGA and GSE39582 datasets. (**B**) The PCR results of common cytokines were confirmed in SW480 cells with control and TIMP1 overexpression. (**C**) Western blot analyses of the indicated proteins in HCT116 and SW480 cells transfected with TIMP1 small interfering or control vector. (**D**) Western blot analyses of the indicated proteins in HCT116 and SW480 cells transfected with TIMP1 overexpression or control vector and treated with ERK1/2 inhibitor 1 (10 nM). (**E**) The migration ability of macrophages was confirmed with Transwell assays in the TIMP1 overexpression group treated with ERK1/2 inhibitor 1 (10 nM). (**F**, **G**) PCR results of detecting the polarization of macrophages under different cocultures with CRC cells treated with ERK1/2 inhibitor 1 (10 nM). (Data are presented as the means ± standard deviations; ^*^indicates *P* < 0.05, ^**^indicates *P* < 0.01, ^***^indicates *P* < 0.001, and ^****^indicates *P* < 0.0001).

We further explored the related mechanism by which TIMP1 regulates macrophages in CRC cells. The functional enrichment analysis found that TIMP1 participated in regulating the ERK1/2 signaling pathway (Figure 6A). Cytokines play important roles in the inflammatory response and intercellular communication. We detected the expression of cytokines in TIMP1-overexpressing SW480 cells by qPCR and found that CCL2, CSF3, CXCL10, CXCL11, ICAM1, IFNG, IL10, IL13 and other cytokines increased significantly, while CSF2, CXCL1, CXCL8 and other cytokines decreased significantly (Figure 6B). Activation of the ERK1/2 signaling pathway can promote the expression of ICAM1 and CCL2. We further found that when TIMP1 was knocked down in HCT116 and SW480 cells, p-ERK1/2, ICAM1 and CCL2 were significantly downregulated, and the total ERK1/2 did not change significantly (Figure 6C). TIMP1 overexpression increased the expression of p-ERK1/2, ICAM1 and CCL2, while total ERK1/2 showed no significant change. Adding ERK1/2 inhibitor 1, an ERK1/2 pathway inhibitor, significantly inhibited the ERK1/2 pathway phosphorylation induced by TIMP1 overexpression in HCT116 and SW480 cells (Figure 6D). In addition, the ERK1/2 inhibitor reduced the expression of ICAM1 and CCL2 caused by TIMP1 overexpression in HCT116 and SW480 cells (Figure 6D). Coculture of HCT116 or SW480 cells overexpressing TIMP1 with macrophages induced by PBMCs significantly promoted the migration of macrophages (Figure 6E), inhibited the expression of M1 markers (CD11C and CD80), and promoted the expression of M2 markers (ARG1 and CD163) (Figure 6F, 6G). The ERK1/2 inhibitor significantly inhibited macrophage migration induced by TIMP1 (Figure 6E) and inhibited the downregulation of M1 markers and the upregulation of M2 markers caused by TIMP1 overexpression in HCT116 and SW480 cells (Figure 6F, 6G).

### The IRG risk score predicted the clinical benefit of immunotherapy

To explore whether the IRG risk score could predict the response to immunotherapy, we applied the IRG risk score in the Van_allen dataset, the sequencing data from metastatic melanoma treatment with cytotoxic T lymphocyte-associated antigen-4 (CTLA-4) blockade [14]. We observed that patients in the low-risk group exhibited a significant clinical benefit in PFS (*p* = 0.023, Supplementary Figure 7B) and a survival benefit trend in OS (*p* = 0.084, Supplementary Figure 7A). The expression analysis revealed that the no clinical benefit group had a higher IRG risk score than the clinical benefit group, but the difference was not significant (Supplementary Figure 7C); meanwhile, the long-term survival group had a higher IRG than the no clinical benefit group. We further discussed the differences in clinical response between the high- and low-risk groups based on the IRG risk score, and the patients in the high-risk group had a lower clinical benefit rate than those in the low-risk group (22.7% vs. 45%, Supplementary Figure 7D). To further explore the relationship between inflammatory response and tumor immunology, a stepwise analysis was performed to reflect the status of anticancer immunity [15]. CRC patients with high TIMP1 expression showed increased activity in all 7 steps of the seven-step Cancer-Immunity Cycle, while TIMP1 expression was mostly correlative with release of cancer cell antigens (Step 1; Pearson, r > 0.4 both in TCGA and GSE39582), trafficking of immune cells to tumors (Step 4; Pearson, r > 0.4 both in TCGA and GSE39582), and infiltration of immune cells into tumors (Step 5; Pearson, r > 0.4 both in TCGA and GSE39582) (Supplementary Figure 8A, 8B). This result indicated that although TIMP1 was helpful for initiation and processing phases of immune response, effective antitumor immunity was still suppressed. Moreover, we discovered that CRC patients with high TIMP1 expression expressed more immune checkpoint molecules than those with low TIMP1 expression (Supplementary Figure 8C, 8D), which was consistent with the “immunity tidal model theory” that high expression of both costimulatory and coinhibitory immune checkpoints caused an immunosuppressive phenotype in tumors [16]. Collectively, the IRG risk score showed predictive power in terms of the clinical benefit of immune checkpoint inhibitors.

## DISCUSSION

Cancer cells live in a network of stromal components comprising fibroblasts, vascular cells, inflammatory cells, and immune cells that interact with each other and together form the TME [3]. These cells in the TME are highly plastic, constantly altering their phenotype and function. Inflammation plays an important role in the composition of the TME, especially in the plasticity of tumor and stromal cells [3]. Hence, we aimed to construct an inflammation-related prognostic model based on the tumor microenvironment to better understand tumor progression and prognosis in CRC.

We constructed a robust inflammation-related prognostic signature comprising eleven IRGs in this study. Some of these eleven IRGs have been reported to be associated with CRC. CCL11 and CCL24 are expressed at lower levels in glandular cells than in stromal cells in CRC tissues, which promotes tumor development. The low expression of CCL11 and CCL24 contributes to immune evasion in CRC because the infiltration of eosinophils is decreased under these conditions [17]. The overexpression of CD36 promotes the progression of solid malignancies such as breast cancer, gastric cancer, glioblastoma and colorectal cancer [18]. SLC4A4 expression in CRC was lower than that in control tissues, and low expression of SLC4A4 was significantly associated with worse survival in CRC [19]. Conversely, TIMP1 is overexpressed in colon cancer and leads to tumor proliferation, metastasis and apoptosis inhibition via the FAK-PI3K/AKT and MAPK pathways [20]. Because a single gene may be unreliable for predicting survival, we constructed an IRG signature in CRC for the first time. The IRG risk score was reliable in stratifying patients with different prognoses in the TCGA and GSE39582 datasets. Survival analyses indicated that the IRG risk score was an independent prognostic index, both as a categorical variable and a continuous variable.

The IRG risk score demonstrated a subtype expression preference that was associated with clinicopathological factors and molecular features in CRC. The relationship between IRGs and clinically meaningful molecular biomarkers, such as BRAF, KRAS and TP53, is notable. The BRAF mutation represents poor prognosis and exhibits resistance to anti-EGFR therapy [21]. Furthermore, the BRAF mutation is associated with the production of CXCL8, a proinflammatory chemokine that can promote tumor proliferation, angiogenesis, and metastasis [22]. In this study, patients with mutant BRAF had relatively higher risk scores than those with wild-type BRAF, suggesting a link between the inflammatory response and BRAF mutation. Moreover, no survival difference was found between wild-type BRAF patients with high IRG risk scores and mutant-type BRAF patients, indicating that the risk associated with IRG may be similar to the risk conferred by the BRAF mutation. Although the mechanism is unclear, genetic or epigenetic alterations in tumors, such as BRAF, KRAS, and TP53 alterations, might influence the inflammatory response status by regulating the expression of these eleven IRGs.

Tumor behavior is not entirely determined by tumor cells alone but rather is also influenced by various nontumor stromal components in the tumor microenvironment, including the extracellular matrix, inflammatory cells, immune cells, mesenchymal cells, fibroblasts, pericytes, and endothelial cells of blood and lymphatic vessels [23]. Functional enrichment analyses demonstrated that the IRGs may modulate the biological processes of the extracellular matrix, cell adhesion, and angiogenesis. The extracellular matrix regulates tissue development and homeostasis, and it influences virtually all behavioral aspects of tumor cells and tumor-associated stromal cells. The extracellular matrix can promote cell proliferation, escape growth inhibition, resist cell death, induce angiogenesis, activate invasion and metastasis, avoid immune destruction and promote chronic inflammation [24, 25]. Integrin mediates interactions with the extracellular matrix and plays roles in matrix synthesis, matrix remodeling, matrix degradation, tumor cell proliferation, tumor stiffness regulation, TGF-β activation, tumor invasion and metastasis [26]. The dysregulation of focal adhesion is a vital determinant of cell migration. Therefore, focal adhesion plays an essential role in promoting the invasion and metastasis of tumor cells [27]. In general, inflammatory response processes accelerate tumor progression and metastasis by disrupting stroma-related biological processes in CRC.

The IRG risk score was positively correlated with the stromal score, suggesting that the inflammatory response participates in regulating stromal components. With the increasing degree of the inflammatory response, fibroblasts, pericytes, M2 macrophages, and MSCs were increasingly enriched in the TME. Abundant studies have proposed the roles of these stromal cells and immune cells in tumor progression and metastasis. Cancer-associated fibroblasts (CAFs) promote the growth, metastasis and diffusion of tumor cells in a variety of ways and hinder the antitumor immune response in the TME [28]. The accumulation of CAFs in the TME is usually related to the poor prognosis of many tumors [28]. Pericytes participate in the formation and maturation of blood vessels and can regulate the degree of immune responses in cancer [29]. MSCs, a type of multipotent stromal cell, can be continuously recruited to tumors and become integral components of the TME, and they are the source of fibroblasts and pericytes [30]. MSCs are also immunoregulatory cells that contribute to effectively suppressing antitumor immunity [31]. Within the tumor, M2 macrophages are a major stromal component that can be recruited into tumor tissues, altering the TME to facilitate tumor progression [32]. This study indicated that the inflammatory response affected stromal components and immune components in the TME, thereby promoting tumor invasion and metastasis. In many cases, more attention has been given to the direct attack on tumor cells, while few studies have been conducted on the microenvironment on which tumor cells rely for survival. Therefore, targeting inflammatory response mediators, such as the IRGs in this signature, is also necessary for effective antitumor therapy.

As the hub gene associated with the inflammatory response in CRC, TIMP1 induces macrophage migration. TIMP-1 is a multifunctional protein that can promote proliferation, growth, and survival, regulate differentiation, and inhibit apoptosis in several tumor types [33]. TIMP1 influences key aspects of the TME, such as by activating cancer-associated fibroblasts, leading to extracellular matrix remodeling, regulating inflammation, stimulating epithelial–mesenchymal transition and influencing angiogenesis, thereby promoting tumor aggressiveness [13, 33]. This study confirmed that TIMP1 is a regulator of intercellular interactions among tumor cells, immune cells, and stromal cells and alters the TME to facilitate tumor development. TIMP1 promotes the expression of ICAM1 and CCL2 by activating the ERK1/2 pathway. ICAM1 and CCL2 can promote macrophage recruitment and phenotypic transformation [34, 35]. Our study confirmed that TIMP1 promotes macrophage migration and mediates macrophage M2 polarization in CRC, and the related mechanism may be that TIMP1 promotes the expression of CLAM1 and CCL2 by activating the ERK1/2 signaling pathway. In the future, identification and obstruction of the tumor-promoting function of TIMP1 may be another method of antitumor treatment.

TMB has gained more and more attention in immunotherapy, which plays an important role in TME and serves as a biomarker for immunotherapy in many types of tumors [36]. Tumors with high TMB are considered to have an increasing burden of new antigens, which makes them immunogenic and sensitive to immunotherapy [37]. In this study, CRC patients with higher IRG risk scores had higher CNV and TMB, which may indirectly predict the efficacy of immunotherapy and guide immunotherapy. Immune checkpoint inhibitors, such as ipilimumab or nivolumab, can induce durable clinical benefits in metastatic colorectal cancer [38, 39]. This study also found that the IRG risk score we constructed could be stratified according to the sensitivity of CTLA-4 inhibitors in metastatic melanoma. Because of no relevant data, the IRG risk scores have not been analyzed for the effect evaluation of immunotherapy for CRC patients, which needs to be validated in CRC in the future. Furthermore, it is still urgent to further investigate the molecular mechanisms of these IRGs involved in the inflammatory response and their prognostic value to support their clinical application.

In conclusion, we constructed and validated a novel IRG risk score as a promising biomarker for CRC individual prognostic assessment and risk stratification. These IRGs participated in extracellular matrix-related pathways and regulated stromal and immune components in the TME; this finding helped to elucidate the underlying mechanism of the inflammatory response in promoting tumors. In the future, targeting these eleven IRGs, especially TIMP1, in combination with anti-stromal therapy or immunotherapy could become a potential therapeutic strategy.

## MATERIALS AND METHODS

### Data acquisition and IRG selection

The RNA sequence and clinical data of 590 CRC samples and 48 colorectal nontumor samples were obtained from the TCGA dataset (https://portal.gdc.cancer.gov/). The gene expression and clinical data in the GSE39582 dataset were obtained from the Gene Expression Omnibus (GEO) (https://www.ncbi.nlm.nih.gov/gds/). The GSE39582 dataset included 581 samples (19 nontumor samples and 562 CRC samples). The Van_allen dataset sequencing from melanoma tumor biopsies pretreated with monoclonal antibodies directed against CTLA-4 was downloaded from the dataset of Genotypes and Phenotypes (dbGaP) (https://www.ncbi.nlm.nih.gov/gap/) [14]. The total inflammation-related genes (IRGs, Supplementary Table 1) included inflammatory response genes (M5932, M10617 and M15261), which are contained in the GSEA dataset (https://www.gsea-msigdb.org/gsea/index.jsp), and inflammasome-related genes [8].

### Cell culture and treatments

The human CRC cell lines HCT116 and SW480 were obtained from ATCC. They were cultured in RPMI-1640 medium containing 10% FBS (Gibco) and 1% penicillin/streptomycin (Gibco) at 37°C with 5% CO_2_. Peripheral blood mononuclear cells (PBMCs) were isolated by centrifugation of human blood with Ficoll-Paque PLUS as previously described [40]. Monocytes were collected by attachment to plastic after 4 h of culture in RPMI-1640 medium containing 10% FBS. ERK1/2 inhibitor (10 nM ERK1/2 inhibitor 1, MedChemExpress, HY-112287, Monmouth Junction, NJ, USA) was used to inhibit the ERK1/2 pathway in HCT116 and SW480 cells.

### Biological phenotypic analysis of macrophages

Cell migration assays were performed to evaluate the motility of macrophages *in vitro*. RT-qPCR was performed to evaluate the polarization of macrophages with CD11C and CD80 for the M1 marker and ARG1 and CD163 for the M2 marker [41].

### Cell transfection

Specific siRNA targeting TIMP1 and a negative control were synthesized by Sangon Biotech (Shanghai, China), and the TIMP1 overexpression plasmid and control vector were purchased from GeneChem Biotech (Shanghai, China). siRNAs and plasmids were transfected with Lipofectamine 3000 reagent (Life Technologies) according to the manufacturer’s instructions. The siRNA sequences of TIMP1 were as follows:

siTIMP1-1 sense (5′–3′): GCACAGUGUUUCCCUGUUUAUTT, antisense (5′–3′): AUAAACAGGGAAACACUGUGCTT.

siTIMP1-2 sense (5′–3′): GAAGUCAACCAGACCACCUUATT, antisense (5′–3′): UAAGGUGGUCUGGUUGACUUCTT.

### Western blotting

CRC cells transfected with knockdown and overexpression of TIMP1 or the corresponding vector were lysed in RIPA buffer, and proteins were collected and denatured. The proteins were subjected to Western blotting analyses as previously described [42]. The proteins were incubated with anti-TIMP1 (PTG, 16644-1-AP), anti-p Erk1/2 (Cell Signaling Technology, 4370), anti-Erk1/2 (Cell Signaling Technology, 4695), anti-CCL2 (Abcam, ab214819), anti-ICAM1 (Abcam, ab282575), or anti-GAPDH (Abcam, ab8245) primary antibodies overnight at 4°C.

### RNA isolation and reverse-transcription qPCR (RT-qPCR)

Total RNA was isolated using TRIzol reagent (Invitrogen, Carlsbad, CA, USA) according to the manufacturer’s instructions. Total RNA was reverse transcribed into cDNA, and qPCR was performed as previously described [42]. The primer sequences are shown in Supplementary Table 6. The mRNA expression of target genes was calculated by the 2^−ΔΔCt^ method and normalized to 18S mRNA expression.

### Macrophage migration assay

For migration assays, PBMC-MØs were digested and resuspended as single cells in serum-free medium. Then, they were seeded into Transwell inserts (Corning, 3422) at a density of 5 × 10^4^ per 200 μL [40]. CRC cells transfected with knockdown and overexpression of TIMP1 or the corresponding vector were seeded in the lower chamber in a 24-well plate with 700 μL medium. After 24 h of incubation, the upper chambers were established as described previously [40].

### Construction of the prognostic model

DEGs among IRGs were analyzed with the “limma” package between tumor samples and normal tissue samples in the TCGA dataset. Adjusted *p* < 0.05 and |log fold change (FC)| > 1.0 were used as cutoff values to select the DEGs. Univariate Cox regression analysis was performed to assess the association between IRG expression and disease outcome in the TCGA dataset. The TCGA dataset contained 21 differentially expressed IRGs with univariate Cox *p* values < 0.05 (Supplementary Table 2). LASSO Cox penalized regression model was performed using the R package “glmnet” to further construct the most significant prognostic model based on the selected IRGs [43]. The coefficients and gene expression values of IRGs were obtained from the LASSO model, and the IRG risk score of each patient was calculated. The formula was as follows:

IRG risk score = ΣIRGs gene expression × coefficient.

### Kaplan-Meier survival analysis and ROC curve analysis

Patients were classified into high-risk and low-risk groups according to the median IRG risk score. The Kaplan-Meier survival curve of each group was plotted using the “survival” R package. Comparing the overall survival, differences with *p* < 0.05 were considered to be significantly different. To verify the accuracy and validity of the IRG risk score, the “pROC” R package was used to calculate the AUC values for 1-, 3- and 5-year OS via ROC analysis [44, 45].

### Construction and verification of the predictive nomogram

A nomogram was constructed with the “rms” R package in the TCGA and GSE39582 datasets [46]. To construct the nomogram, univariate Cox regression analysis of clinical data and the IRG risk score was performed. Pathological stage, pathological T stage, pathological N stage, pathological M stage and IRG risk score had *p* < 0.05 in the univariate analysis, and they were integrated into the predictive nomogram. Then, a calibration curve was generated, and the C-index was calculated to test the prediction accuracy of the nomogram. A calibration curve was used to evaluate whether the predicted value of the model was consistent with the occurrence probability of the outcome [47]. The C-index evaluated the probability that the predicted results were consistent with the actual observed results. The pathological stage, pathological T stage, pathological N stage, pathological M stage, IRG risk score and nomogram score were compared through C-index analysis.

### Somatic mutation and somatic CNV data analysis

CNV profile and somatic mutation data were collected from the TCGA dataset (https://portal.gdc.cancer.gov/). GISTIC2.0 was used to calculate CNV associated with the IRG risk score. A GISTIC value less than -1 or more than 1 was defined as gene deletion or gene amplification, respectively. The “maftools” R package was used to visually analyze the somatic mutation data based on VarScan software.

### GO analysis and KEGG pathway analysis

IRG risk score related genes were used to perform the GO and KEGG analysis through the website Database for Annotation, Visualization and Integrated Discovery (DAVID) 6.8 (https://david.ncifcrf.gov/tools.jsp) in the TCGA or GSE39582, respectively [48].

### GSEA and GSVA

GSEA (https://www.gsea-msigdb.org/gsea/index.jsp) was performed to explore significantly different biological processes between the high-risk and low-risk groups. The false discovery rate (FDR) and normalized enrichment score (NES) were used to determine the statistical significance [49]. GSVA (http://www.bioconductor.org) was used to further verify the correlation between the IRG risk score and GSVA value on the basis of the gene sets of defined signaling pathways [50].

### Tumor purity, stromal score, immune score, and cell population enrichment analysis

Tumor purity, stromal score and immune score were calculated with the ESTIMATE method as previously described [44]. For the cell population enrichment analysis, 64 immune and stromal cell types were evaluated by xCell [12].

### WGCNA construction and hub gene identification

The “WGCNA” package in R software was utilized to construct the gene coexpression network to identify the inflammatory response-related hub genes in CRC. First, an appropriate soft threshold power was selected to construct a scale-free topology module. Next, the network interconnectivity was built by TOM, and gene modules were identified based on the hierarchical clustering method. Then, module-trait correlations were tested by Pearson’s correlation analysis between each module eigengene (ME) and each clinical characteristic (IRG risk score, pathological stage, pathological T stage, pathological N stage, and pathological M stage) to identify module members. The module with the highest correlation coefficient was identified as the key module. GS represents the relationship between genes and traits, and MM represents the relationship between MEs and gene expression profiles. Finally, we selected the gene with the highest GS to be the hub gene in the key module.

### Statistical analysis

Statistical analysis was mostly based on GraphPad Prism 7 software. *P* value < 0.05 was defined as statistically significant. Significant quantitative differences between and among groups were calculated by one-way ANOVA and two-tailed *t* test, respectively. Kaplan-Meier survival analysis was performed with R (version 3.6.2). The log-rank test was used to evaluate the differences between stratified groups. Univariate and multivariate Cox regression analyses were used to estimate the prognostic value of the IRG risk score.

## Supplementary Materials

Supplementary Figures

Supplementary Table 1

Supplementary Table 2

Supplementary Tables 3, 4 and 6

Supplementary Table 5
