# Supplementary material for: A novel inflammation-related signature for predicting prognosis and characterizing the tumor microenvironment in colorectal cancer
Source: Aging (Albany NY). 2023 Apr 2;15(7):2554–81. doi: 10.18632/aging.204630 (PMC10120913; doi:10.18632/aging.204630)
Supplement: Supplementary Figures [file aging-15-204630-s001.pdf]

SUPPLEMENTARY FIGURES

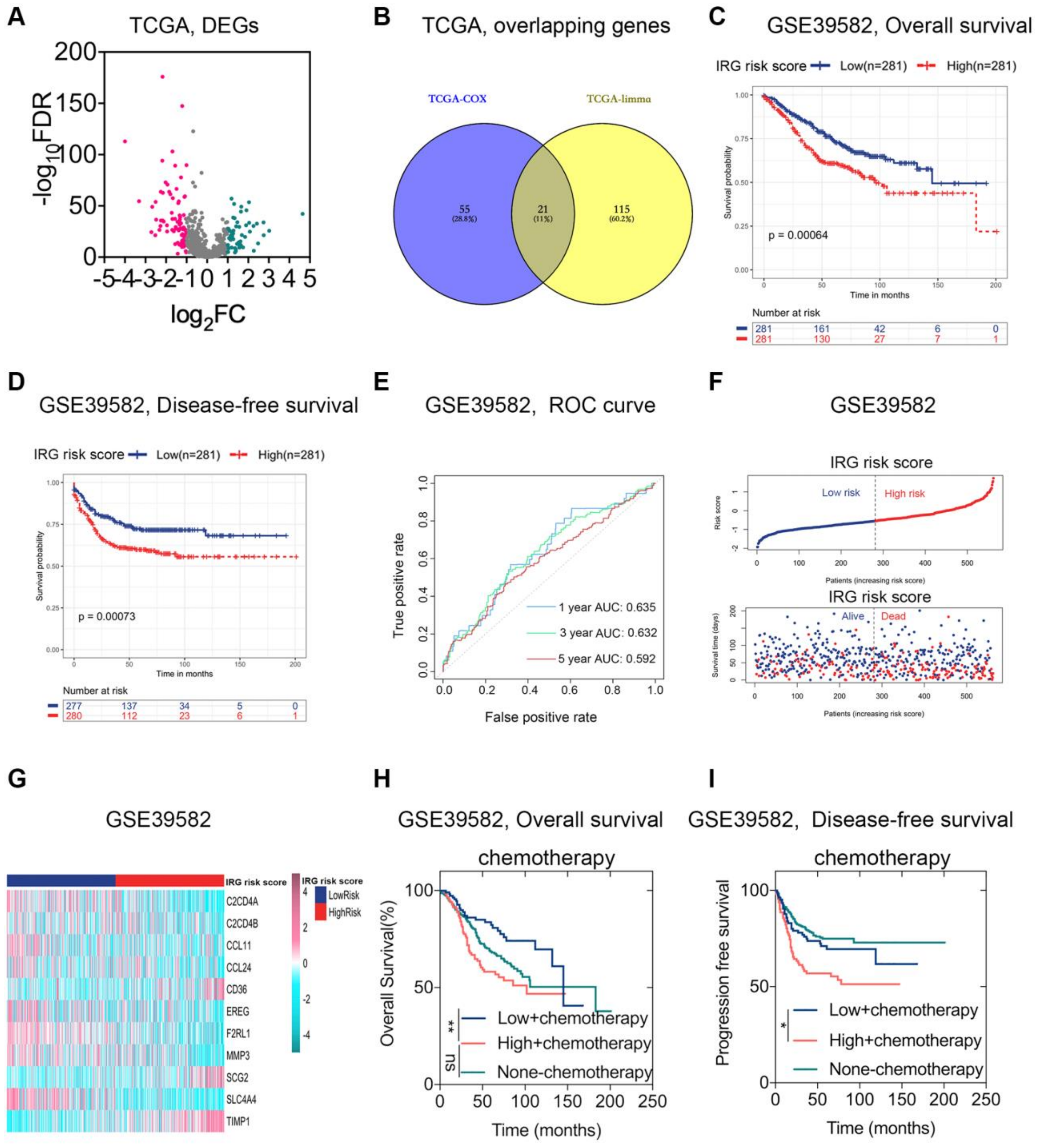

Supplementary Figure 1. Construction and validation of the IRG risk score in CRC. (**A**) Differential expression of IRGs in CRC tissue samples in the TCGA dataset. (**B**) The overlapping IRG genes of DEGs and prognostic genes based on univariate Cox regression analysis. (**C**) Kaplan-Meier analysis of overall survival (OS) based on the IRG signature of CRC patients in the GSE39582 database (log-rank *P* value = 0.00064). (**D**) Kaplan-Meier analysis of disease-free survival (DFS) based on the IRG signature of CRC patients in the GSE39582 database (log-rank *P* value = 0.00073). (**E**) Receiver operating characteristic (ROC) curve of the IRG signature for 1-, 3- and 5-year overall survival in the GSE39582 database. (**F**, **G**) The distribution of risk scores, survival status and gene expression data of CRC patients based on the IRG signature in the GSE39582 database. (**H**, **I**) Kaplan-Meier analysis of overall survival and disease-free survival (DFS) in subgroups stratified by both IRG signature and receipt of adjuvant chemotherapy.

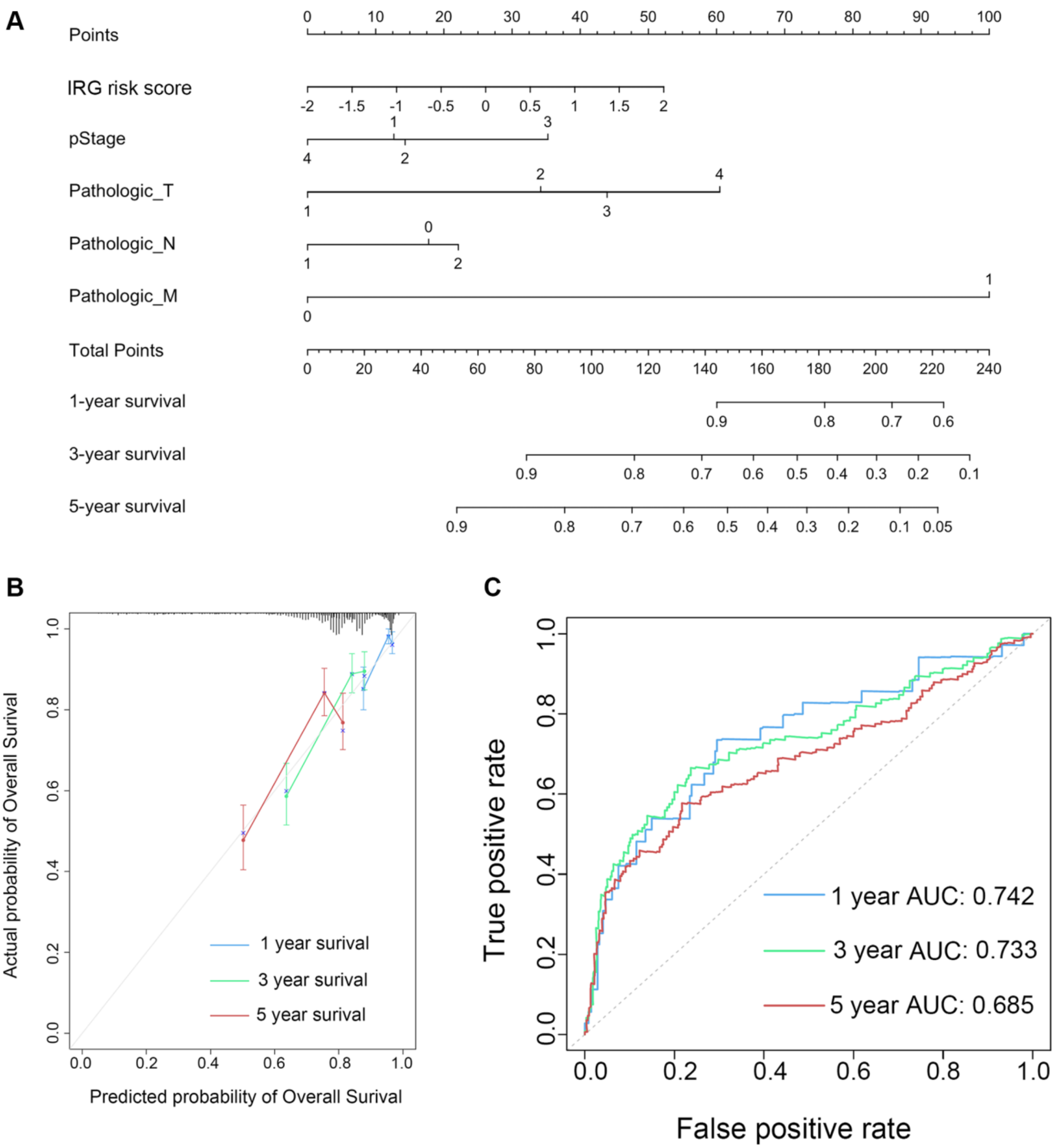

Supplementary Figure 2. Development and validation of the IRG risk score nomogram in GSE39582. (**A**) Development of the IRG signature nomogram in GSE39582. (**B**) The calibration plot exhibited wonderful agreement between prediction and observation in the probabilities of 1-, 3- and 5-year overall survival. (**C**) Receiver operating characteristic (ROC) curve of the IRG nomogram for 1-, 3- and 5-year overall survival in the GSE39582 database.

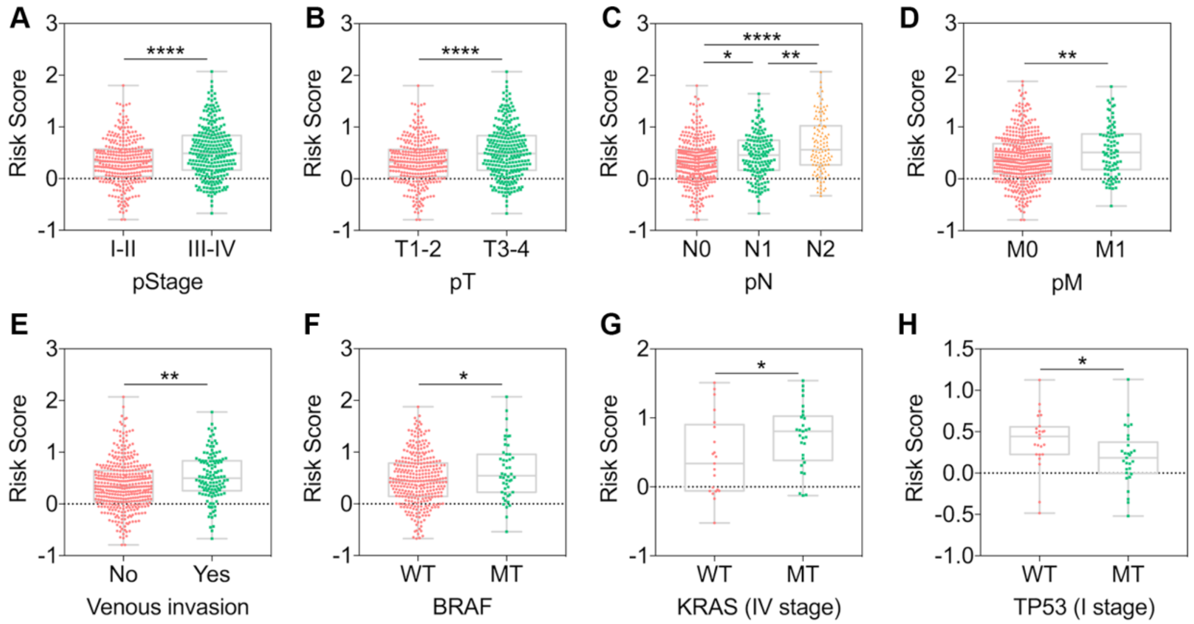

Supplementary Figure 3. The IRG risk score showed different expression values in different cohorts. (**A**–**H**) Distribution of risk scores of the IRG signature according to tumor TNM stage, pT stage, pN stage, pM stage, venous invasion, and molecular type in the TCGA dataset.

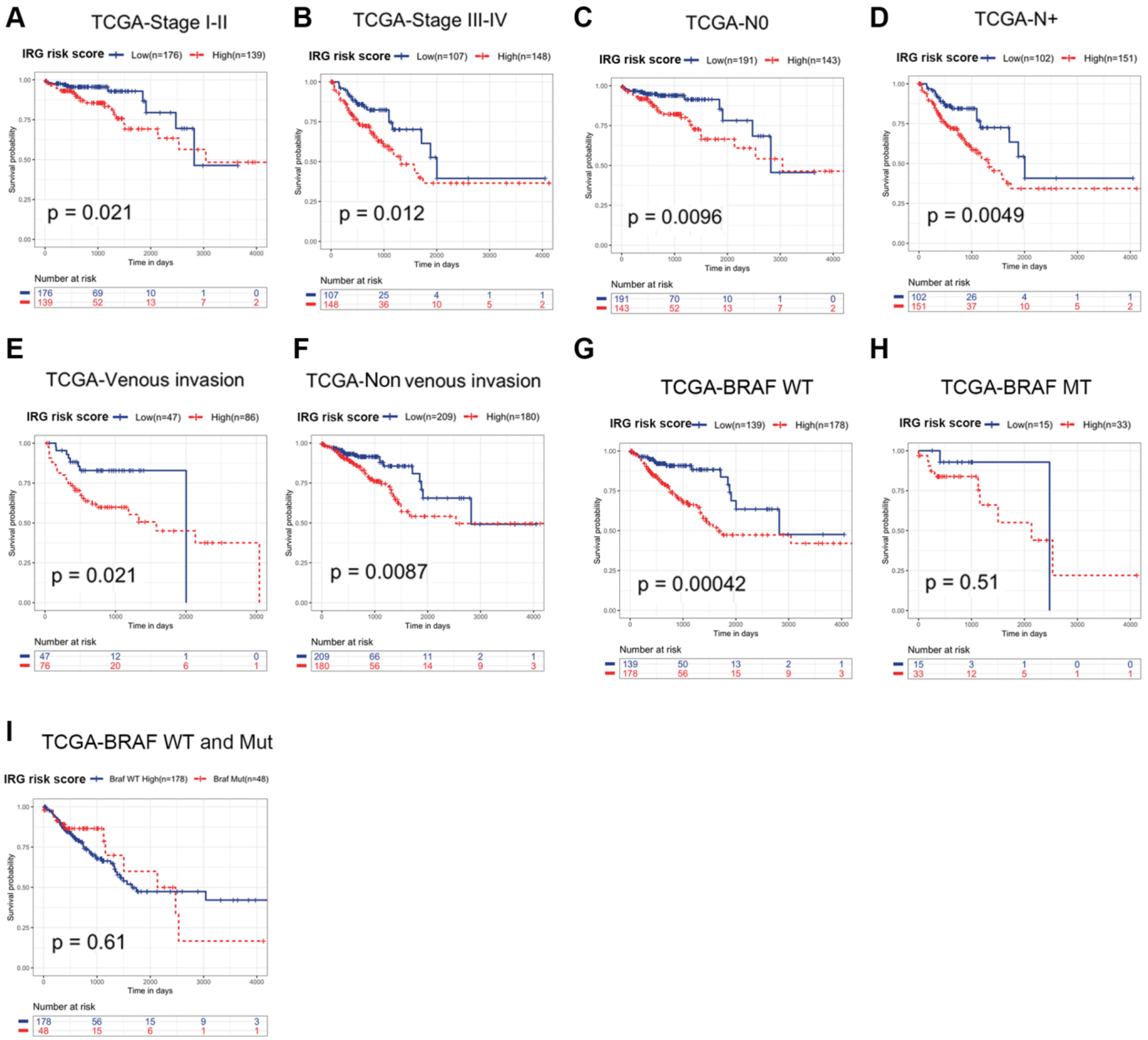

Supplementary Figure 4. The IRG risk score showed different prognostic values in different cohorts. (**A**–**H**) The IRG signature showed significant prognostic value in different cohorts stratified by tumor TNM stage, pN stage, venous invasion, and BRAF type in the TCGA dataset through the log-rank test for trend. (**I**) Survival comparison between wild-type BRAF patients with high scores and mutant-type BRAF patients in the TCGA dataset.

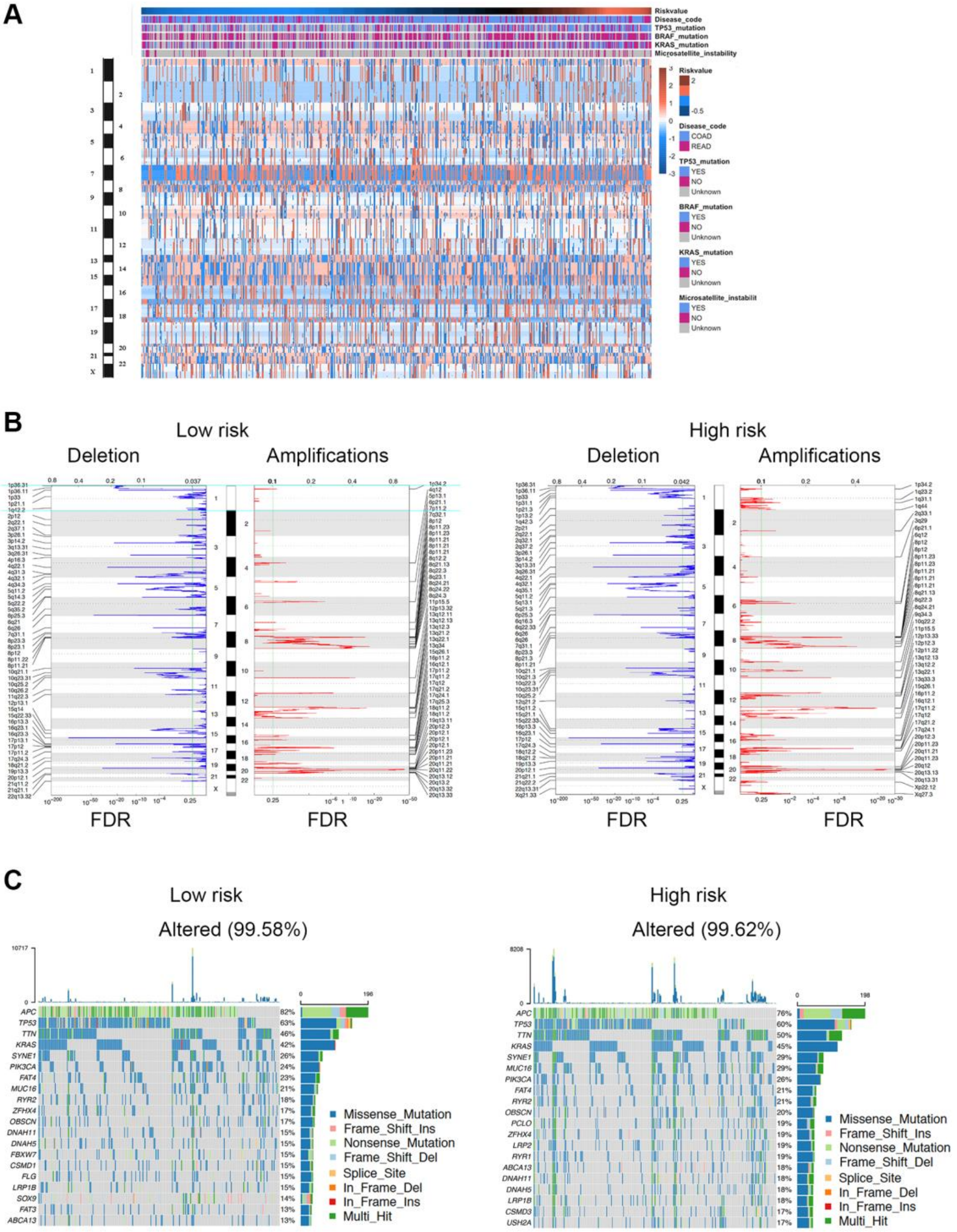

Supplementary Figure 5. Specific somatic mutation and somatic copy number alteration in different IRG risk scores. (**A**) CNV spectrum with ascending order of the IRG risk score. (**B**) A distinguishing CNV spectrum was observed between the low- and high-risk score groups. The incidence of chromosome deletion (blue) and chromosome amplification (red) are presented on the horizontal axis. (**C**) The waterfall plot of tumor somatic mutations established by the low- and high-risk score groups. Each column represents each patient. The upper bar plot shows TMB. The number on the right represents the mutation frequency of each gene. The right bar plot shows the proportion of each variant type.

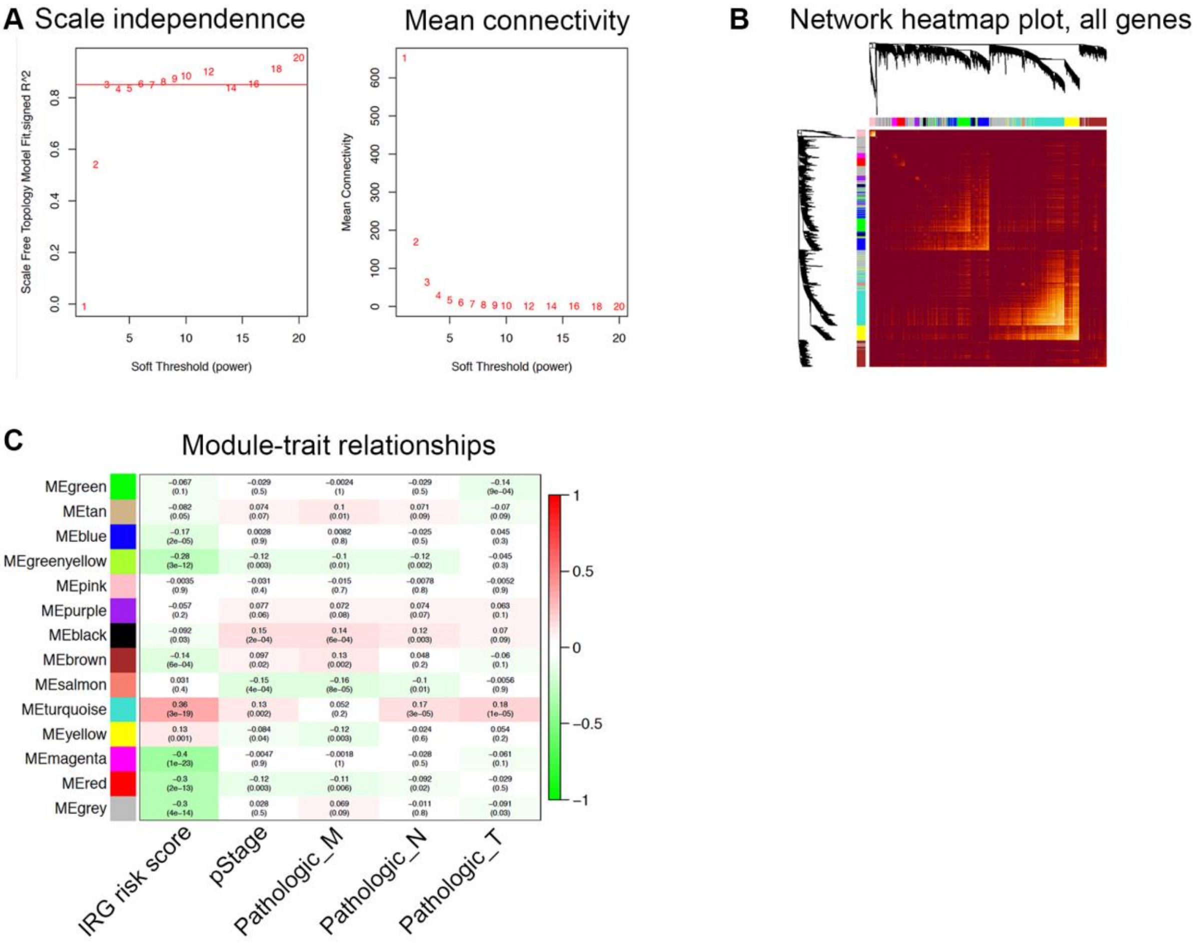

Supplementary Figure 6. TIMP1 was the hub gene of the inflammatory response in CRC. (**A**) Soft threshold analysis. Each power corresponded to scale independence and mean connectivity. A soft threshold of 3 (red horizontal line) was chosen for the construction of the scale-free topology module. (**B**) Heatmap described the TOM among 4575 selected genes in WGCNA. A lighter color represents higher overlap, and a darker color corresponds to lower overlap. (**C**) Module-trait relationships between module eigengenes and clinical traits (IRG risk score, pathological stage, pathological T stage, pathological N stage, and pathological M stage). The correlation coefficient, *p* value and correlation coefficient were shown in each block.

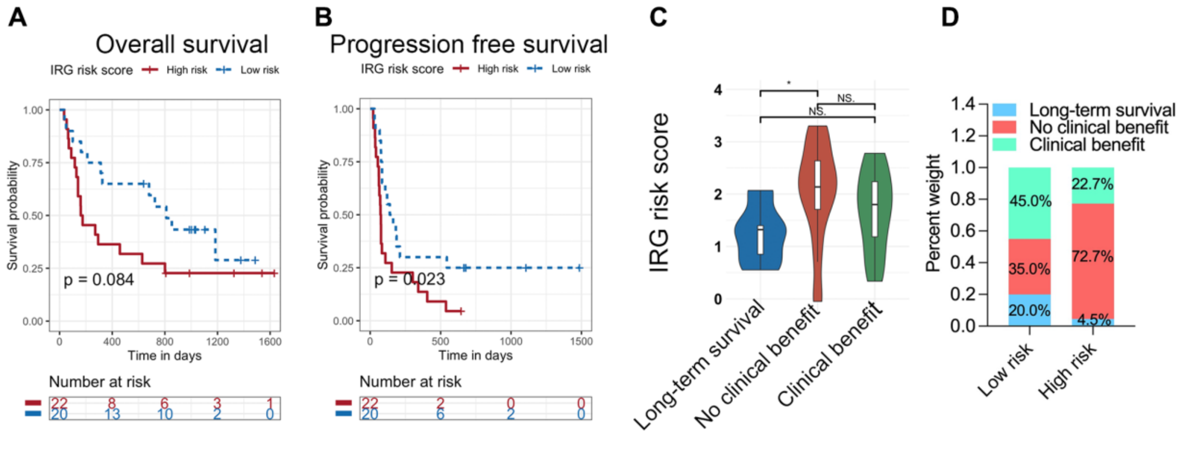

Supplementary Figure 7. The IRG risk score predicted the therapeutic response to immunotherapy. (**A**, **B**) Kaplan-Meier analysis of overall survival (**A**) and progression-free survival (**B**) based on the IRG risk score of metastatic melanoma patients treated with ipilimumab in the Van_allen dataset. (**C**) Distribution of the IRG risk score according to different groups in the Van Allen datasets. (**D**) The proportion of different patient responses in the low- or high-risk groups in the Van_allen dataset.

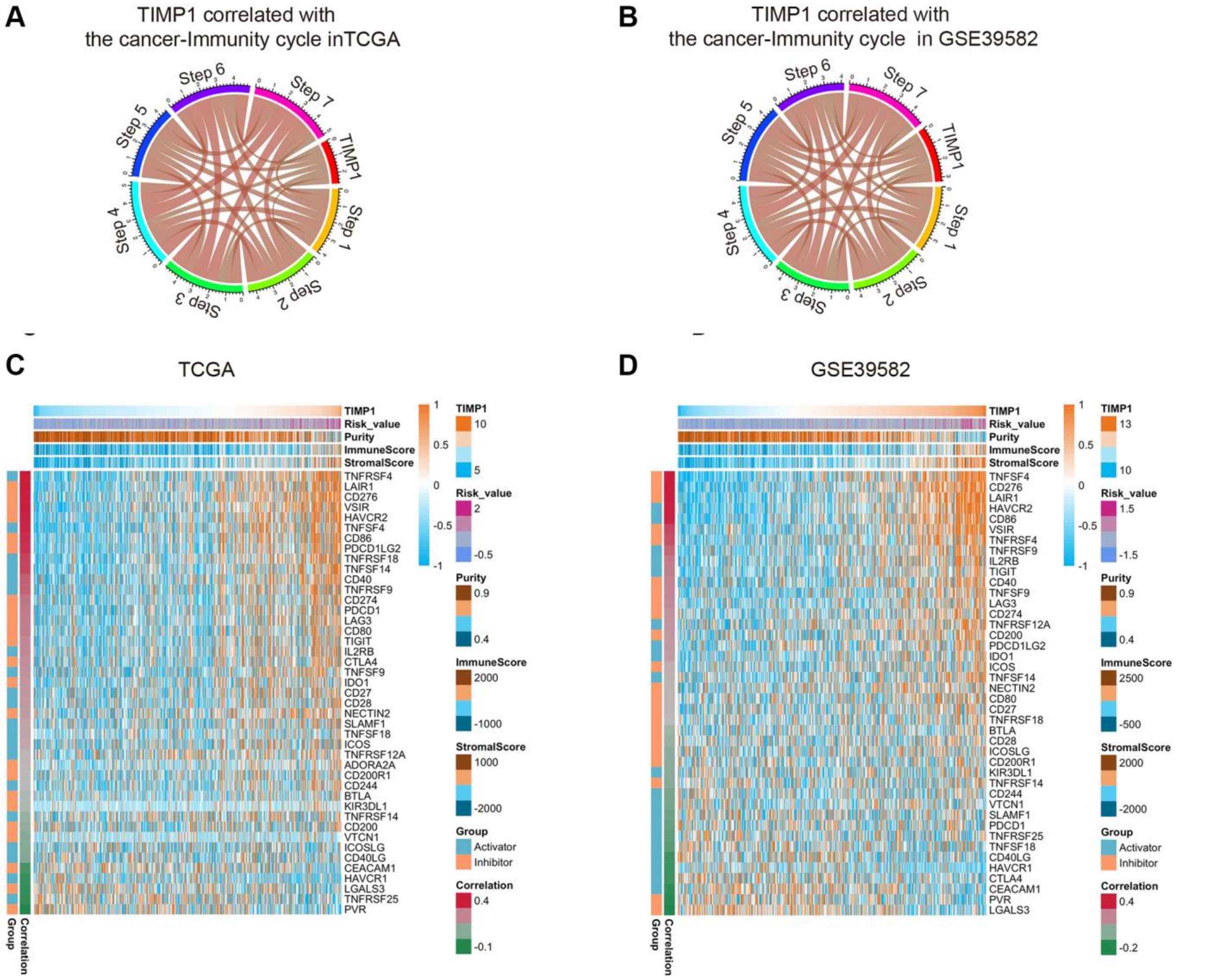

Supplementary Figure 8. TIMP1 was associated with the immunosuppressive microenvironment in the TCGA and GSE39582 datasets. (**A**, **B**) Correlation between TIMP1 and various steps of the cancer immunity cycle in the TCGA (**A**) and GSE39582 (**B**) datasets. (**C**, **D**) The differential expression of immune checkpoint molecules with increased TIMP1 expression in the TCGA (**C**) and GSE39582 (**D**) datasets.
