# Supplementary material for: A novel inflammation-related signature for predicting prognosis and characterizing the tumor microenvironment in colorectal cancer
Source: Aging (Albany NY). 2023 Apr 2;15(7):2554–81. doi: 10.18632/aging.204630 (PMC10120913; doi:10.18632/aging.204630)
Supplement: Supplementary Table 1 [file aging-15-204630-s002.docx]

**Supplementary Table 1. List of IRGs.**

| **Gene** | **Gene** | **Gene** | **Gene** | **Gene** |
| --- | --- | --- | --- | --- |
| AIM2 | IL18 | SEMA7A | CYBB | RIPK2 |
| CARD8 | IL1B | SERPINE1 | CYP26B1 | RPS6KA4 |
| CASP1 | IL1R1 | SERPINF1 | CYP4F11 | RPS6KA5 |
| CASP12 | IL1R2 | SETD6 | CYSLTR1 | S1PR3 |
| CASP4 | IL1RL1 | SHARPIN | DAB2IP | SAA2 |
| CASP5 | IL1RL2 | SHPK | DPEP1 | SAA4 |
| DDX3X | IL2 | SIGLEC10 | ECM1 | SCG2 |
| DHX33 | IL20 | SIRPA | EIF2AK1 | SCN9A |
| GSDMD | IL20RB | SLAMF8 | ELF3 | SCUBE1 |
| NAIP | IL21 | SLC7A2 | EPHA2 | SCYL1 |
| NLRC4 | IL22RA2 | SMAD3 | EPO | SCYL3 |
| NLRP1 | IL23A | SMPDL3B | F11R | SDC1 |
| NLRP3 | IL2RA | SNCA | F2R | SEH1L |
| NLRP6 | IL33 | SNX4 | F2RL1 | SELP |
| NLRP9 | IL37 | SOCS3 | F3 | SERPINA1 |
| PYCARD | IL4 | SOCS5 | F8 | SERPINA3 |
| APP | IL6 | SOD1 | FN1 | SERPINC1 |
| BCL2 | IL6ST | SPATA2 | FOLR2 | SERPINF2 |
| BCL2L1 | INS | SPHK1 | FOS | SGMS1 |
| HSP90AB1 | IRF3 | STAP1 | FPR1 | SIGIRR |
| MEFV | ISL1 | STAT3 | FPR3 | SIGLEC1 |
| NFKB1 | ITGA2 | STAT5B | GAL | SLC11A1 |
| NFKB2 | JAK2 | STING1 | GGT5 | SMAD1 |
| P2RX7 | KARS1 | STK39 | GJA1 | SMO |
| PANX1 | KLF4 | SUCNR1 | GPR32 | SNAP23 |
| PSTPIP1 | KLKB1 | SYT11 | GPR32P1 | SPP1 |
| RELA | KPNA6 | TAC1 | GPR33 | STAB1 |
| SUGT1 | KRT1 | TAFA3 | GPR68 | STARD7 |
| TXN | LACC1 | TBC1D23 | H2BC1 | SYK |
| TXNIP | LBP | TEK | HAMP | TAC4 |
| ABCC1 | LDLR | TGM2 | HAVCR2 | TACR1 |
| ABCD1 | LEP | TICAM1 | HDAC4 | TBK1 |
| ABCD2 | LILRA5 | TLR10 | HDAC5 | TBXA2R |
| ABHD12 | LPCAT3 | TLR2 | HDAC9 | TCIRG1 |
| ABR | LPL | TLR3 | HFE | TFR2 |
| ACE2 | LRFN5 | TLR4 | HIF1A | TGFB1 |
| ACOD1 | LRRC19 | TLR6 | HK1 | THBS1 |
| ACP5 | LRRK2 | TLR7 | HMGB1 | THEMIS2 |
| ADA | LTA | TLR9 | HMGB2 | TICAM2 |
| ADAM8 | LYN | TMSB4X | HMOX1 | TIMP1 |
| ADAMTS12 | MACIR | TNF | HNRNPA0 | TIRAP |
| ADCY7 | MAPK13 | TNFAIP3 | HP | TLR1 |
| ADCYAP1 | MAPK14 | TNFAIP6 | HPR | TLR5 |
| ADIPOQ | MAPK7 | TNFAIP8L2 | HRH1 | TLR8 |
| ADORA1 | MCPH1 | TNFRSF11A | HRH4 | TMIGD3 |
| ADORA2A | MDK | TNFRSF1A | HSPG2 | TNFRSF4 |
| ADORA2B | METRNL | TNFRSF1B | HYAL1 | TNIP2 |
| AGER | MFHAS1 | TNFSF11 | HYAL3 | TNIP3 |
| AGT | MGLL | TNFSF4 | ICAM1 | TOLLIP |
| AGTR1 | MGST2 | TNIP1 | IFI16 | TPST1 |
| AHSG | MIR105-1 | TRADD | IFNA2 | TREM1 |
| AKNA | MIR105-2 | TREM2 | IFNGR1 | TRIL |
| ALOX15 | MIR125A | TREX1 | IGFBP4 | TRPV1 |
| ALOX5 | MIR126 | TRPV4 | IKBKB | TSPAN2 |
| ANXA1 | MIR128-1 | TSLP | IKBKG | TUSC2 |
| AOAH | MIR128-2 | TTBK1 | IL10RB | TYROBP |
| APCS | MIR129-1 | TYRO3 | IL17C | UCN |
| APOA1 | MIR129-2 | UFL1 | IL17RE | UGT1A1 |
| APOD | MIR135A1 | USP18 | IL18R1 | UNC13D |
| APOE | MIR136 | VAMP7 | IL18RAP | VNN1 |
| APPL1 | MIR138-1 | VAMP8 | IL1A | WDR83 |
| APPL2 | MIR138-2 | VPS35 | IL1F10 | XCL2 |
| AREL1 | MIR140 | WFDC1 | IL1RAP | XCR1 |
| ASH1L | MIR141 | WNT5A | IL1RN | ZNF580 |
| ATM | MIR142 | XCL1 | IL22 | ABCA1 |
| BAP1 | MIR144 | XIAP | IL23R | ABI1 |
| BCL6 | MIR145 | ZBP1 | IL25 | ACVR1B |
| BCL6B | MIR146A | ZC3H12A | IL27 | ACVR2A |
| BCR | MIR149 | ZP3 | IL31RA | ADGRE1 |
| BIRC2 | MIR15A | ZYX | IL34 | ADRM1 |
| BIRC3 | MIR16-1 | ABCF1 | IL36A | AHR |
| BRD4 | MIR16-2 | ACER3 | IL36B | APLNR |
| BST1 | MIR17 | ACKR1 | IL36G | AQP9 |
| BTK | MIR181A1 | ACKR2 | IL36RN | ATP2A2 |
| C1QTNF12 | MIR181A2 | ACVR1 | IL4R | ATP2B1 |
| C1QTNF3 | MIR181B1 | ADCY1 | IL5 | ATP2C1 |
| C2CD4A | MIR181B2 | ADCY8 | IL5RA | BEST1 |
| C2CD4B | MIR181C | ADGRE2 | IL6R | BST2 |
| C3 | MIR187 | ADGRE5 | IL9 | BTG2 |
| CALCRL | MIR195 | ADM | IRAK2 | CD48 |
| CCL1 | MIR197 | ADORA3 | IRGM | CD55 |
| CCL24 | MIR19A | AFAP1L2 | ITCH | CD69 |
| CCL3 | MIR19B1 | AGTR2 | ITGAL | CD70 |
| CCL5 | MIR19B2 | AHCY | ITGAM | CD82 |
| CCN3 | MIR203A | AIF1 | ITGB2 | CDKN1A |
| CCN4 | MIR204 | AIMP1 | ITGB6 | CLEC5A |
| CCR2 | MIR205 | AKT1 | ITIH4 | CSF3 |
| CCR7 | MIR206 | ANO6 | JAM3 | CSF3R |
| CD200 | MIR20A | AOC3 | JUN | DCBLD2 |
| CD200R1 | MIR21 | AP3B1 | KDM6B | EBI3 |
| CD200R1L | MIR22 | APOA2 | KIT | EDN1 |
| CD28 | MIR221 | APOL2 | KLRG1 | EIF2AK2 |
| CD47 | MIR222 | APOL3 | KNG1 | EMP3 |
| CD6 | MIR223 | ASS1 | KRT16 | EREG |
| CD81 | MIR26A1 | ATRN | LAT | FZD5 |
| CDH5 | MIR26A2 | AXL | LGALS9 | GABBR1 |
| CDK19 | MIR30C2 | AZU1 | LIAS | GCH1 |
| CEBPA | MIR31 | B4GALT1 | LIPA | GNA15 |
| CEBPB | MIR338 | BACE2 | LOXL3 | GNAI3 |
| CHID1 | MIR361 | BDKRB1 | LRP1 | GP1BA |
| CLEC7A | MIR378A | BDKRB2 | LTB4R | GPC3 |
| CLOCK | MIR3909 | BLNK | LTB4R2 | GPR132 |
| CMA1 | MIR488 | BMP2 | LXN | GPR183 |
| CNR1 | MIR590 | BMP6 | LY75 | HAS2 |
| CNR2 | MIR657 | BMPR1B | LY86 | HBEGF |
| CREB3L3 | MIR6869 | C1QA | LY96 | HPN |
| CST7 | MIR766 | C3AR1 | LYZ | ICAM4 |
| CTSC | MIR920 | C4A | MAP2K3 | ICOSLG |
| CUEDC2 | MIR92A1 | C4B | MAPKAPK2 | IFITM1 |
| CX3CL1 | MIR92A2 | C5 | MAPT | IFNAR1 |
| CXCL17 | MIR93 | C5AR1 | MBL2 | IFNGR2 |
| CYLD | MIR98 | C5AR2 | MEP1B | IL10RA |
| CYP19A1 | MIRLET7G | CALCA | MIF | IL15RA |
| DAGLA | MMP26 | CAMK1D | MMP25 | IL2RB |
| DAGLB | MMP3 | CAMK4 | MRGPRX1 | IL7R |
| DDT | MMP8 | CARD18 | MS4A2 | INHBA |
| DEFB114 | MMP9 | CCL11 | MSMP | IRF1 |
| DHX9 | MVK | CCL13 | MYLK3 | IRF7 |
| DNASE1 | MYD88 | CCL14 | NAMPT | ITGA5 |
| DNASE1L3 | NAPEPLD | CCL15 | NCR3 | ITGB3 |
| DROSHA | NDFIP1 | CCL16 | NDST1 | ITGB8 |
| DUOXA1 | NEAT1 | CCL17 | NFAM1 | KCNA3 |
| DUOXA2 | NFE2L1 | CCL18 | NFATC3 | KCNJ2 |
| DUSP10 | NFKBIA | CCL19 | NFATC4 | KCNMB2 |
| EDNRB | NFKBIZ | CCL2 | NFE2L2 | KIF1B |
| EGFR | NLRC3 | CCL20 | NFKBID | KLF6 |
| ELANE | NLRP10 | CCL21 | NFRKB | LAMP3 |
| ENPP3 | NLRP12 | CCL22 | NFX1 | LCK |
| ESR1 | NLRP7 | CCL23 | NLRP2 | LCP2 |
| ETS1 | NLRX1 | CCL25 | NLRP4 | LIF |
| F12 | NOD2 | CCL26 | NMI | LPAR1 |
| F2 | NOS2 | CCL3L1 | NOD1 | LY6E |
| FABP4 | NPPA | CCL3L3 | NOTCH1 | MARCO |
| FANCA | NPY5R | CCL4 | NOTCH2 | MEP1A |
| FANCD2 | NR1D1 | CCL7 | NOX1 | MET |
| FCER1G | NR1D2 | CCL8 | NOX4 | MMP14 |
| FCGR2B | NR1H3 | CCR1 | NPFF | MSR1 |
| FEM1A | NR1H4 | CCR3 | NRROS | MXD1 |
| FFAR2 | NT5E | CCR4 | ODAM | MYC |
| FFAR3 | NUPR1 | CCR5 | OGG1 | NDP |
| FFAR4 | OSM | CCR6 | OLR1 | NMUR1 |
| FNDC4 | OSMR | CCRL2 | OPRM1 | NPFFR2 |
| FOXF1 | OTULIN | CD14 | ORM1 | OPRK1 |
| FOXP1 | PARK7 | CD163 | ORM2 | P2RX4 |
| FOXP3 | PBK | CD180 | P2RX1 | P2RY2 |
| FPR2 | PDCD4 | CD36 | PARP4 | PCDH7 |
| FUT7 | PDE2A | CD40 | PF4 | PDE4B |
| GATA3 | PER1 | CD40LG | PF4V1 | PDPN |
| GBA | PGLYRP1 | CD44 | PIK3CD | PIK3R5 |
| GBP5 | PGLYRP2 | CD5L | PJA2 | PLAUR |
| GGT1 | PIK3AP1 | CD68 | PLA2G2E | PTPRE |
| GGT2 | PIK3CG | CD96 | PLA2G4B | PVR |
| GGT3P | PLA2G2A | CDO1 | PLA2G4C | RAF1 |
| GHRL | PLA2G2D | CELA1 | PLAA | RGS1 |
| GHSR | PLA2G3 | CERS6 | PLGRKT | RGS16 |
| GPER1 | PLA2G7 | CHI3L1 | PLP1 | RHOG |
| GPR17 | PLD3 | CHIA | PLSCR1 | RNF144B |
| GPR31 | PLD4 | CHST1 | PNMA1 | ROS1 |
| GPR4 | PPARA | CHST2 | POLB | RTP4 |
| GPRC5B | PPARD | CHST4 | PPBP | SCARF1 |
| GPS2 | PPARG | CHUK | PRCP | SCN1B |
| GPSM3 | PRKCD | CIITA | PRDX2 | SELL |
| GPX1 | PROC | CLU | PRDX5 | SEMA4D |
| GPX4 | PSMA1 | CMKLR1 | PRKCQ | SGMS2 |
| GRN | PSMA6 | CNTF | PRKCZ | SLAMF1 |
| GSTP1 | PSMB4 | CRH | PRKD1 | SLC11A2 |
| HCK | PTGER3 | CRHBP | PROK2 | SLC1A2 |
| HGF | PTGER4 | CRP | PSEN1 | SLC28A2 |
| HLA-DRB1 | PTGIS | CSF1 | PTAFR | SLC31A1 |
| HLA-E | PTGS2 | CSF1R | PTGDR | SLC31A2 |
| HSPD1 | PTPN2 | CSRP3 | PTGER1 | SLC4A4 |
| HYAL2 | PTPRC | CTNNBIP1 | PTGER2 | SLC7A1 |
| IDO1 | PYDC2 | CXCL1 | PTGES | SRI |
| IFNG | RB1 | CXCL10 | PTGFR | TACR3 |
| IGF1 | RHBDD3 | CXCL11 | PTGIR | TAPBP |
| IL10 | RICTOR | CXCL13 | PTGS1 | TNFRSF9 |
| IL12B | RIPK1 | CXCL2 | PTN | TNFSF10 |
| IL13 | RORA | CXCL3 | PTX3 | TNFSF15 |
| IL15 | RPS19 | CXCL5 | PXK | TNFSF9 |
| IL16 | S100A12 | CXCL6 | RAC1 | TPBG |
| IL17A | S100A8 | CXCL8 | RARRES2 | VIP |
| IL17B | S100A9 | CXCL9 | RASGRP1 | NLRC5 |
| IL17D | SAA1 | CXCR2 | RBPJ | DDX58 |
| IL17F | SBNO2 | CXCR3 | REG3A |  |
| IL17RA | SCGB1A1 | CXCR4 | REG3G |  |
| IL17RB | SELE | CXCR6 | REL |  |
| IL17RC | SELENOS | CYBA | RELB |  |
