# Supplementary material for: A novel inflammation-related signature for predicting prognosis and characterizing the tumor microenvironment in colorectal cancer
Source: Aging (Albany NY). 2023 Apr 2;15(7):2554–81. doi: 10.18632/aging.204630 (PMC10120913; doi:10.18632/aging.204630)
Supplement: Supplementary Tables 3, 4 and 6 [file aging-15-204630-s004.pdf]

SUPPLEMENTARY TABLES

Supplementary Table 3. Univariate Cox regression analyses of clinical information with the overall survival of CRC patients.

**TCGA dataset**			
**Variable**	**Univariate regression**		
	**HR**	**95% CI**	***p* Value**
Risk score	3.8334	2.3288–6.3101	0.0000
Pathologic_stage	2.0305	1.5438–2.6708	0.0000
Pathologic_T	2.5614	1.5665–4.1880	0.0002
Pathologic_N	1.9038	1.4319–2.5313	0.0000
Pathologic_M	3.2602	1.9388–5.4824	0.0000
TP53_mutation	1.8464	1.0562–3.2278	0.0314
KRAS_mutation	0.8982	0.5611–1.4381	0.6549
BRAF_mutation	1.2109	0.6353–2.3080	0.5608
**GSE39582 dataset**			
**Variable**	**Univariate regression**		
	**HR**	**95% CI**	***p* Value**
Risk score	1.5919	1.1676–2.1702	0.0033
Pathologic_stage	1.9919	1.5290–2.5949	0.0000
Pathologic_T	1.7872	1.2300–2.5968	0.0023
Pathologic_N	1.5973	1.2601–2.0248	0.0001
Pathologic_M	4.6461	2.9194–7.3941	0.0000
TP53_mutation	1.2812	0.8645–1.8987	0.2171
KRAS_mutation	1.2408	0.8407–1.8315	0.2773
BRAF_mutation	1.5943	0.8515–2.9852	0.1449

Supplementary Table 4. Univariate and multivariate Cox regression analyses of the IRG risk score in GSE39582 datasets with the overall survival of CRC patients.

**GSE39582 dataset**						
**Variable**	**Univariate regression**			**Multivariate regression**		
	**HR**	**95% CI**	***p* Value**	**HR**	**95% CI**	***p* Value**
Risk score	1.5919	1.1676–2.1702	0.0033	1.3746	1.0119–1.8670	0.0418
Pathologic_stage	1.9919	1.5290–2.5949	0	0.9004	0.4943–1.6400	0.7319
Pathologic_T	1.7872	1.2300–2.5968	0.0023	1.2692	0.8539–1.8860	0.2384
Pathologic_N	1.5973	1.2601–2.0248	0.0001	1.3186	0.8702–0.8702	0.1922
Pathologic_M	4.6461	2.9194–7.3941	0	3.8438	1.6796–8.7970	0.0014

Supplementary Table 6. The RT-qPCR primer sequences and antibodies were used in this article.

**The RT-qPCR primer sequences**		
**Gene**	**Forward primer**	**Reverse primer**
ITGAX (CD11c)	AGAGCTGTGATAAGCCAGTTCC	AATTCCTCGAAAGTGAAGTGTGT
CD80	GGCCCGAGTACAAGAACCG	TCGTATGTGCCCTCGTCAGAT
ARG1	GTGGAAACTTGCATGGACAAC	AATCCTGGCACATCGGGAATC
CD163	TTTGTCAACTTGAGTCCCTTCAC	TCCCGCTACACTTGTTTTCAC
TIMP1	ACCACCTTATACCAGCGTTATGA	GGTGTAGACGAACCGGATGTC
18S	GCAGAATCCACGCCAGTACAAGAT	TCTTCTTCAGTCGCTCCAGGTCTT
ARPC1B	CAAGGACCGCACCCAGATT	TGCCGCAGGTCACAATACG
C5	ACAGTCATAGAGTCTACAGGTGG	CCAACTGGTCAAGCGAATCTT
CCL1	CTCATTTGCGGAGCAAGAGAT	GCCTCTGAACCCATCCAACTG
CCL2	AGAATCACCAGCAGCAAGTGTCC	TCCTGAACCCACTTCTGCTTGG
CCL3	AGTTCTCTGCATCACTTGCTG	CGGCTTCGCTTGGTTAGGAA
CCL4	CTGTGCTGATCCCAGTGAATC	TCAGTTCAGTTCCAGGTCATACA
CCL5	CCTGCTGCTTTGCCTACATTGC	ACACACTTGGCGGTTCTTTCGG
CD40LG	ACATACAACCAAACTTCTCCCCG	GCAAAAAGTGCTGACCCAATCA
CXCL1	AGCTTGCCTCAATCCTGCATCC	TCCTTCAGGAACAGCCACCAGT
CXCL10	GGTGAGAAGAGATGTCTGAATCC	GTCCATCCTTGGAAGCACTGCA
CXCL11	GACGCTGTCTTTGCATAGGC	GGATTTAGGCATCGTTGTCCTTT
CXCL12	ATTCTCAACACTCCAAACTGTGC	ACTTTAGCTTCGGGTCAATGC
G-CSF	GCTGCTTGAGCCAACTCCATA	GAACGCGGTACGACACCTC
GM-CSF	TCCTGAACCTGAGTAGAGACAC	TGCTGCTTGTAGTGGCTGG
ICAM1	ATGCCCAGACATCTGTGTCC	GGGGTCTCTATGCCCAACAA
IFNG	GAGTGTGGAGACCATCAAGGAAG	TGCTTTGCGTTGGACATTCAAGTC
IL10	TCTCCGAGATGCCTTCAGCAGA	TCAGACAAGGCTTGGCAACCCA
IL12	CCTTGCACTTCTGAAGAGATTGA	ACAGGGCCATCATAAAAGAGGT
IL13	CCTCATGGCGCTTTTGTTGAC	TCTGGTTCTGGGTGATGTTGA
IL16	GCCGAAGACCCTTGGGTTAG	GCTGGCATTGGGCTGTAGA
IL17a	TCCCACGAAATCCAGGATGC	GGATGTTCAGGTTGACCATCAC
IL18	TCTTCATTGACCAAGGAAATCGG	TCCGGGGTGCATTATCTCTAC
IL1a	TGGTAGTAGCAACCAACGGGA	ACTTTGATTGAGGGCGTCATTC
IL1b	CCACAGACCTTCCAGGAGAATG	GTGCAGTTCAGTGATCGTACAGG
IL1ra	CATTGAGCCTCATGCTCTGTT	CGCTGTCTGAGCGGATGAA
IL2	AACTCCTGTCTTGCATTGCAC	GCTCCAGTTGTAGCTGTGTTT
IL21	TAGAGACAAACTGTGAGTGGTCA	GGGCATGTTAGTCTGTGTTTCTG
IL25	CAGGTGGTTGCATTCTTGGC	GAGCCGGTTCAAGTCTCTGT
IL27/	ACCGCTTTGCGGAATCTCA	AGGTCAGGGAAACATCAGGGA
IL32a	TGGCGGCTTATTATGAGGAGC	CTCGGCACCGTAATCCATCTC
IL4	CCAACTGCTTCCCCCTCTG	TCTGTTACGGTCAACTCGGTG
IL5	TGGAGCTGCCTACGTGTATG	TTCGATGAGTAGAAAGCAGTGC
IL6	AGACAGCCACTCACCTCTTCAG	TTCTGCCAGTGCCTCTTTGCTG
IL8	ACTGAGAGTGATTGAGAGTGGAC	AACCCTCTGCACCCAGTTTTC
SerpineE1	ACCGCAACGTGGTTTTCTCA	TTGAATCCCATAGCTGCTTGAAT
TNFa	CCTCTCTCTAATCAGCCCTCTG	GAGGACCTGGGAGTAGATGAG
TREM1	GAACTCCGAGCTGCAACTAAA	TCTAGCGTGTAGTCACATTTCAC
ATF3	CGCTGGAATCAGTCACTGTCAG	CTTGTTTCGGCACTTTGCAGCTG
IRF2	CATGCGGCTAGACATGGGTG	GCTTTCCTGTATGGATTGCCC

CEBPA	AGGAGGATGAAGCCAAGCAGCT	AGTGCGCGATCTGGAACTGCAG
CEBPB	CTTCAGCCCGTACCTGGAG	GGAGAGGAAGTCGTGGTGC

The antibodies

**Antibody**	**Identifier**
Anti-p Erk1/2	4370
Anti-Erk1/2	4695
Anti-CCL2	ab214819
Anti-ICAM1	ab282575
Anti-TIMP1	16644-1-AP
Anti-GAPDH	ab8245
ERK1/2 inhibitor 1	HY-112287
